# Identification of PANoptosis-related predictors for prognosis and tumor microenvironment by multiomics analysis in glioma

**DOI:** 10.7150/jca.94200

**Published:** 2024-03-11

**Authors:** Fengzeng Sun, Miaomiao Liao, Zi Tao, Ruiqi Hu, Jun Qin, Weiwei Tao, Wentong Liu, Yiqi Wang, Guoliang Pi, Junrong Lei, Wendai Bao, Zhiqiang Dong

**Affiliations:** 1 College of Biomedicine and Health, College of Life Science and Technology, Huazhong Agricultural University, Wuhan, China.; 2 Center for Neurological Disease Research, Taihe Hospital, Hubei University of Medicine, Shiyan, China.; 3 Department of Neurosurgery, Taihe Hospital, Hubei University of Medicine, Shiyan, China.; 4 Central Laboratory, Hubei Cancer Hospital, Wuhan, China.; 5 Department of Radiation Oncology, Hubei Cancer Hospital, Wuhan, China.

**Keywords:** Glioma, PANoptosis, Tumor immune microenvironment, Prognostic model, MYBL2, TUBA1C

## Abstract

PANoptosis is a newly described inflammatory programmed cell death, that highlights coordination between pyroptosis, apoptosis and necroptosis. However, the functions of PANoptosis-related genes in glioma progression still remain to be explored. This study aims to identify PANoptosis-related predictors that may be utilized for prognosis prediction and development of new therapeutic targets. Firstly, bulk and single-cell RNA-seq (scRNA-seq) data of glioma patients were extracted from TCGA, CGGA and GEO database. Genetic analysis indicates a considerably high mutation frequency of PANoptosis-related genes (PANRGs) in glioma. Consensus clustering was applied to reveal different subtypes of glioma based on PANRGs. Two PANoptosis subtypes with distinct prognostic and TME characteristics were identified. Then, with LASSO-Cox regression analysis, four PANoptosis-related predictors (MYBL2, TUBA1C, C21orf62 and KCNIP2) were determined from bulk and scRNA-seq analysis. Predictive PANRG score model was established with these predictors and its correlation with tumor microenvironment (TME) was investigated. The results showed that patients with low PANRG score, had higher infiltration of anti-tumor immune cells, higher MSI score and lower TIDE score, which are more likely to benefit from immunotherapy. Further analysis identified 16 potential drugs associated with PANoptosis-related predictors. Moreover, the expression levels of four PANoptosis-related predictors were examined in clinical samples and the results were consistent with those analyzed in the database. Besides, we also confirmed the biological functions of two oncogenic predictors (MYBL2 and TUBA1C) by cell experiments, which revealed that knockdown of MYBL2 or TUBA1C could significantly inhibit the proliferation and migration of glioma cells. These findings highlight the prognostic value and biological functions of PANRGs in glioma, which may provide valuable insights for individualized treatment.

## Introduction

Glioma is the most common type of primary tumor in the central nervous system 1. The grades of gliomas were defined according to the classification of the World Health Organization (WHO) 2, more than half of glioma patients suffer from glioblastoma multiforme (GBM, grade IV), which are fatal and incurable 3, 4. Despite decades of therapy were development for this disease, including lesion excision assisted with radiotherapy and chemoradiotherapy with temozolomide (TMZ) 5, 6, the recurrence of gliomas is still inevitable. Considering the high heterogeneity in molecular characteristics 7 and brain tumor microenvironment (TME) for gliomas 3, 8, there is an urgent need for the establishment of an accurate and reliable predictive model for diagnosis and treatments.

Resistance to cell death is one of the hallmarks of cancer 9. PCD is a controlled process required for the elimination of unwanted cells including cancerous and infected cells. Thus, the regulation of PCD cascade is closely related to the pathogenesis and progression of tumors, including gliomas 10, 11. Apoptosis, pyroptosis, and necroptosis are the most genetically well-defined PCD pathways 12. Recent studies have found extensive crosstalk between these pathways, and established the concept of PANoptosis, a newly described inflammatory PCD pathway with key features of these three PCD forms that cannot be accounted for any of these pathways alone 13-15. Activation of PANoptosis has shown to be beneficial in treatments for certain cancers, such as colorectal cancer and melanoma 16-18. In recent years, pyroptosi-, apoptosis- and necroptosis-related genes were found to be dysregulated during the pathological process of glioma 19-21. Nevertheless, the pathogenic role and regulatory mechanisms of PANoptosis in glioma remains elusive. Therefore, a comprehensive analysis of the roles of PANoptosis in glioma progression may facilitate the understanding of underlying mechanism in glioma tumorigenesis and guiding individualized treatment.

This study focuses on the identification of PANoptosis associated predictors that can accurately predict overall survival (OS) and TME characteristics. In total, 200 PANRGs were collected from multiple databases and preceding publications. The genetic landscape of PANRGs was significantly altered in glioma samples. Based on the expression of PANRGs, the glioma patients were divided into two subtypes, which showed distinct prognosis and TME characteristics. Subsequently, we developed a prognostic PANRG score for predicting the OS outcome of glioma patients with genes from bulk-seq and scRNA-seq analysis. The PANRG score was also significantly correlated with TME characteristics and potential drug responses. In addition, we verified the expression and function of PANoptosis-associated predictors in clinical samples and glioma cells, which might be a promising therapeutic target for the treatment of glioma.

## Material and Methods

### Data source and processing

RNA sequencing (RNA-seq) data and corresponding clinical information for patients with glioma were extracted from The Cancer Genome Atlas (TCGA, https://portal.gdc.cancer.gov/) and the Chinese Glioma Genome Atlas (CGGA, http://www.cgga.org.cn/) 22-25. A total of 1136 glioma samples were enrolled in this study (Table S1). The somatic mutation data was collected from the TCGA database, analyzed with R package maftools 26. The CNV burdens were defined as the number of genes lying within CNVs in each sample 27. The Microsatellite Instability (MSI) were acquired as previously reported 28.

The scRNA-seq data (GSE173278) of glioma was downloaded from the GEO database and analyzed with R package Seurat 29, 30. In total, 26,299 single cells of 10 primary glioma patients were enrolled in this study. Cell annotation was processed with R package SingleR and manual cell type annotation 31. The R package inferCNV was used to distinguish malignant tumor cells from non-tumor cells 32. A PANoptosis score was calculated with PANoptosome genes 13, 33-37 which construct a molecular platform triggering PANoptosis by using the “PercentageFeatureSet” function.

### Clinical patient samples and cell lines

Ten glioma tissue samples (5 WHO grade II/ III and 5 WHO grade IV) were obtained from the Neurosurgery Department of Taihe Hospital (Hubei University of Medicine, Shiyan, China) with informed consent. The study was approved by the Medical Ethics Committee of Taihe Hospital (Approval number: 2023KS10).

Human glioma cells (U251, LN229) were provided by Cell Bank, Chinese Academy of Sciences. All cells were cultivated in DMEM medium (HyClone, USA), supplemented with 10% fetal bovine serum (Gibco, USA) and cultured in a standard 3 ℃ incubator.

### Identification of PANRGs

Genes associated with pyroptosis, apoptosis and necroptosis 12 were included as PANoptosis related genes (PANRGs) in our study. In total, 200 PANRGs were extracted from MsigDB (REACTOME_PYROPTOSIS) and previous publications 38-41 (Table S2).

The protein-protein interaction (PPI) network of PANRGs was constructed with STRING database 42 and rebuilt by Cytoscape 43.

### Consensus Clustering

The R package “ConsensusClusterPlus” was employed for consensus unsupervised clustering analysis according to the expression levels of PANRGs 44. Kaplan-Meier (K-M) survival analysis was performed to evaluate the overall survival (OS) of patients in two PANoptosis subtypes.

### Differential Gene expression and Functional enrichment analysis

The differentially expressed genes (DEGs) between two subtypes were screened by using the limma package in R 45.

Functional enrichment analysis, including Gene Ontology (GO) 46 and Kyoto Encyclopedia of Genes and Genomes (KEGG) pathway 47 analysis, were performed using R package clusterProfiler. Gene set variation analysis (GSVA) was performed with the MSigDB hallmark (c2.cp.kegg.v7.2) 48. For bulk-seq data, ssGSEA analysis was conducted to calculate the PANoptosis score based on PANoptosome genes 13, 33-37.

### Assessment of the immunological characteristics of the TME

ESTIMATE algorithm was employed to evaluate the immune and stromal components 49. The infiltration level of 22 tumor-infiltrating immune cells (TIICs) was quantified by CIBERSORTx algorithm 50.

### Construction and Validation of the prognostic PANRG score model

Prognostic PANRGs associated with OS were first screened according to subtype-related DEGs by using univariate Cox regression analysis. Lasso Cox regression algorithm was then utilized to diminish the scope of the prognosis-related genes with penalty parameter tuning performed by 10-fold cross-validation implemented in R package glmnet. Finally, genes with non-zero LASSO regression coefficients were included in multivariate Cox regression analysis.

The PANRG score constructed by Lasso Cox regression algorithm was calculated as follows:



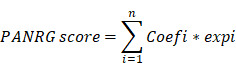



Where Coef and exp denote the coefficient and expression of each gene, respectively.

### Establishment and Evaluation of predictive nomogram

Univariate and multivariate Cox regression analyses were conducted to assess whether the risk score could be regarded as an independent variable factor while considering other clinical features 51. A predictive nomogram was established with the R package rms. The Calibration and Time-dependent ROC curves were utilized to evaluate the accuracy of the nomogram for OS prediction. Besides, we performed Decision curve analysis (DCA) to estimate the survival net benefits.

### Prediction of immunotherapy response

Gene expression profiles of glioma samples were subjected to the TIDE algorithm to predict the potential immune checkpoint blockade (ICB) therapy response.

### Potential Compounds Prediction

Estimated IC50 of chemotherapy drugs were evaluated based on the GDSC database by applying the R package pRRophetic 52, 53. CMap database (https://clue.io/) was used to explore potential drugs 54.

### Small interfering RNA (siRNA) mediated genes knockdown

Small interfering RNAs against MYBL2 and TUBA1C were from Genecreate (Wuhan, China). Transfection was performed with the Lipofectamine 3000 Transfection Kit (Thermo Fisher, USA) according to the manufacturer's instructions. The sequences of the siRNAs were listed in Supplementary Table S3.

### Western blotting

Cells were lysed using RIPA buffer (Beyotime, China) supplemented with a protease inhibitor cocktail (Beyotime). The cellular lysates were centrifuged at 13,000 rpm for 10 min. The protein concentrations were determined using a BCA protein assay kit (Beyotime) according to the manufacturer's instructions. Equal number of proteins (20 μg) were separated by SDS-PAGE, and the separated proteins were transferred onto 0.45 μm PVDF membranes (Merck Millipore, Ireland). After the membrane was sealed with 5% skim milk, the corresponding protein bands were cut out according to the marker and then incubated overnight at 4 °C in the corresponding primary antibodies listed in Table S4. A super-sensitive chemiluminescence (ECL) kit (Beyotime, China) was used to detect chemiluminescence signals.

### Real-time quantitative PCR

Total RNA was extracted with TRIzol reagent (Invitrogen, USA). cDNA was synthesized by reverse transcription with PrimeScript™ RT reagent kit (Takara, Japan) according to the manufacturer's instructions. RT-qPCR was performed to detect the expression of the four PANoptosis-related predictors (MYBL2, C21orf62, TUBA1C and KCNIP2) by using TB Green® Premix Ex Taq™ II kit (Takara) on a CFX96 Real-Time PCR Detection System (Bio-Rad, USA). The primers used for RT-qPCR are summarized in Supplementary Table S5.

### Cell viability assay

Cell viability was detected by using the Cell Counting Kit-8 (CCK8, Dojindo, Japan) following the manufacturer's protocol. In brief, an equal number of transfected cells were seeded into 96-well plates. Absorbance was measured at a wavelength of 450 nm. EdU assay was also performed to assess cell proliferation with EdU Cell Proliferation Kit (Beyotime), following the manufacturer's instructions.

### Cell Migration Assays

Cell migration was assessed via transwell and wound healing assay. For transwell assay, glioma cells were transfected with siRNA or negative control for 48 h. Transfected cells in 100μL serum free media were added into the upper chamber, 500μL culture media with 20% FBS was in the bottom chamber. migrated cells on the lower surface of the membrane were stained with 0.1% crystal violet. Five fields were randomly selected for counting the number of migrated cells, and images were taken by a microscopy.

Wound healing assay was performed as follow. Briefly, transfected cells were plated on a 6-well plate at 1.5 × 10^6^ cells per well. Twelve hours after plating, scratches were made using 10 μl pipette tips. Forty-eight hours later, cells were visualized by light microscopy. Each experiment was repeated three times.

### Statistical Analysis

The comparisons of normally distributed variables between groups were analyzed by the paired Student's t-test and unpaired Student's t-test. The comparisons of non-normally distributed variables between groups were analyzed by the Mann-Whitney *U* test. The Kruskal-Wallis test was used to compare multiple groups. Experiments were performed in triplicate. The statistical analyses in this study were implemented with R 4.1.0 software. P-value *<*0.05 was considered statistically significant.

## Results

### Characterization of PANRGs in glioma

**Figure 1:**
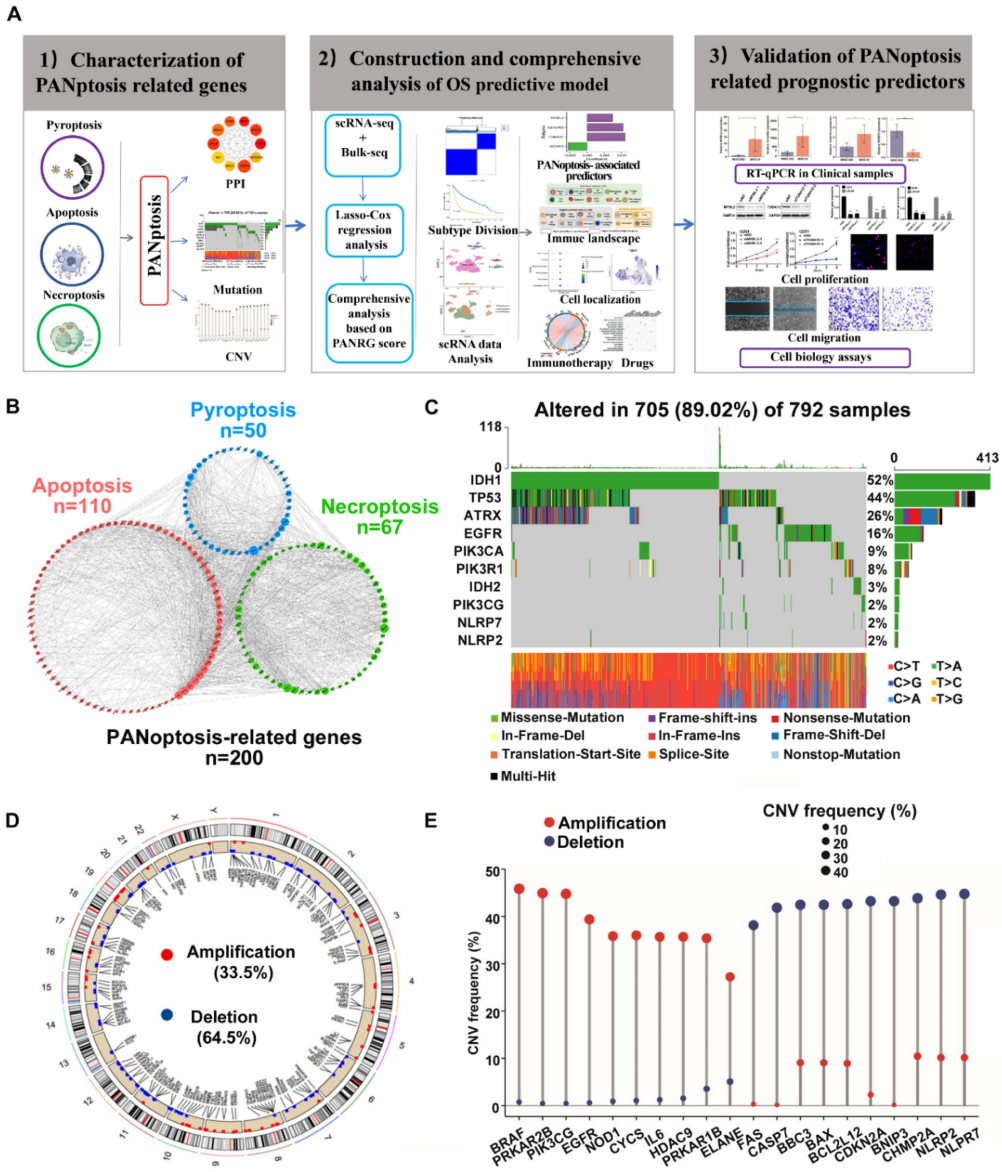
Characterization of PANRGs in glioma (A)Schematic illustration of the study design. (B) PPI network of the 200 PANRGs. (C) PANRGs with Top-10 mutation frequencies in glioma patient. (D) Frequencies and chromosome locations of CNV gain, loss, and non-CNV among PANRGs. (E) Frequencies of CNV amplification, deletion, and non-CNV among representative PANRGs.

The overall design is shown in Figure 1A. This study included a total of 200 PANRGs collected from various database and previous studies (Figure 1B, Figure S1A). To explore the internal connections between all these PANRGs, we conducted a PPI analysis, 10 of which were identified as hub genes, which may play a key role in the regulation of PANoptosis (Figure 1B; Figure S1B, C and Table S6).

Genetic alterations analysis of these genes showed a considerably high mutation frequency of PANRGs in glioma. Among the 792 samples, 89.02% (705) had mutations in the PANRGs (Figure 1C). Four genes (IDH1, TP53, ATRX, EGFR) possessed relatively highest mutation frequency. Next, we further analyzed the somatic copy number of PANRGs in glioma. The results showed that prevalent CNVs could be observed in most of the PANRGs (196/200) (Figure 1D, E). Among them, 67 genes (33.5%) had significant amplifications, while 129 genes (64.5%) had significant deletions. The locations of CNV alterations in the PANRGs were also displayed (Figure 1D). These results showed that the genetic landscape of PANRGs were significantly altered in glioma samples, which implicates key roles of PANoptosis pathways in glioma.

### Identification of two PANoptosis subtypes with distinct prognostic, molecular and TME features

**Figure 2:**
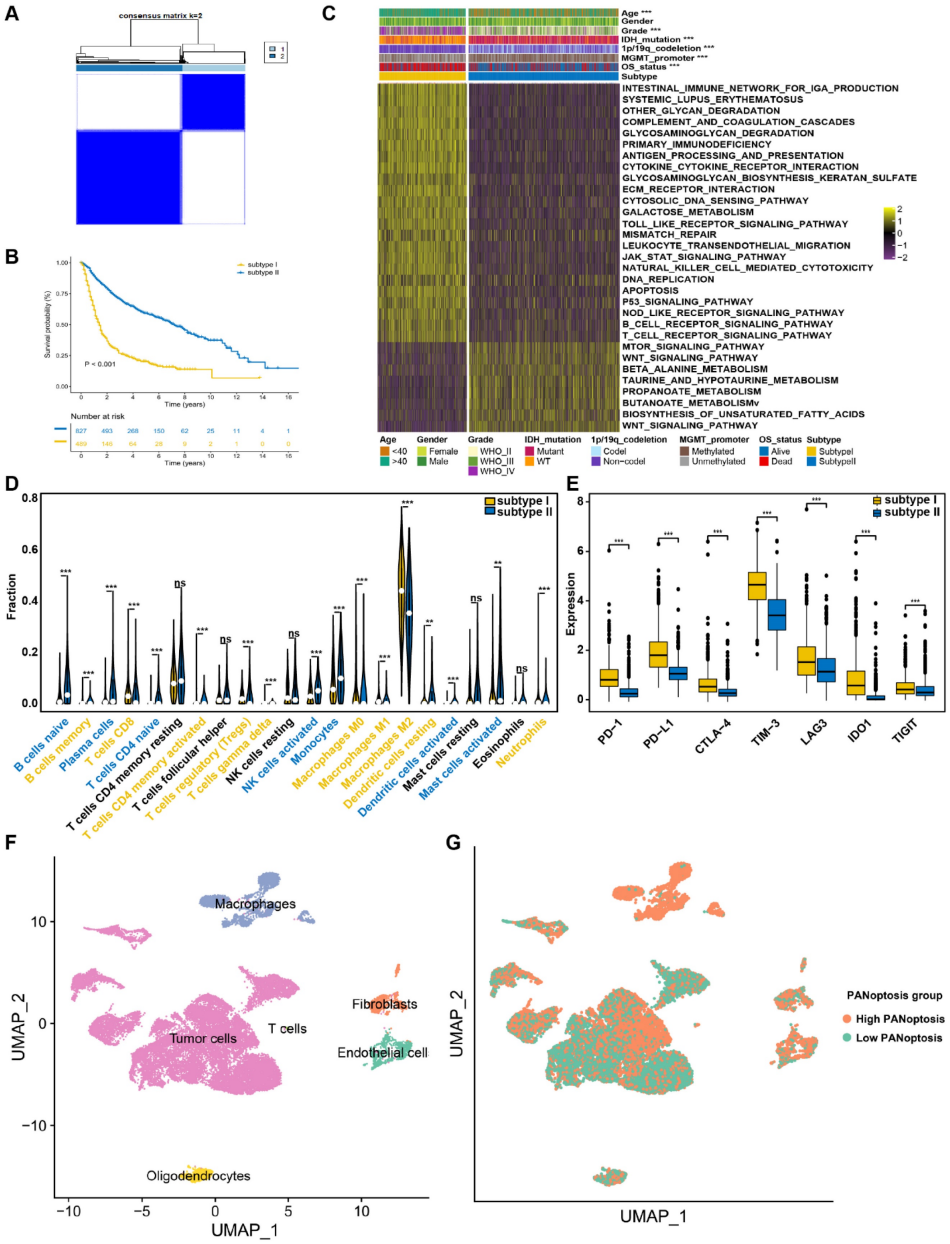
Identification of two PANoptosis subtypes with distinct survival outcomes, clinical features and TME characteristics (A) Consensus clustering matrix for k = 2. (B) Survival analysis for patients between two subtypes in TCGA dataset. The log-rank test was used to determine the statistical significance of the differences, and P < 0.05 was considered significant. (C) GSVA analysis and clinicopathologic features between PANoptosis subtypes. (D) Abundance of 22 types of TIICs between PANoptosis subtypes. (E) The expression levels of immune checkpoints. * P < 0.05, ** P < 0.01, *** P < 0.001. (F) Annotation of cell types in glioma. (G) Scatter plot of high- and low-PANoptosis cells distinguished with different colors.

To uncover the expression characteristics of PANRGs in glioma, we used a consensus clustering algorithm to categorize the glioma patients based on the expression of the PANRGs. The consensus matrix (CM), CDF curves and delta area were calculated to identify the optimal cluster number (n = 2) (Figure 2A, Figure S2A-C). Then, patients were divided into two distinct subtypes: I (n = 489) and II (n = 827). PCA analysis revealed significant differences in PANoptosis transcription profiles between these two subtypes (Figure S2D). The results of prognostic analysis demonstrated that subtype II had an OS advantage over subtype I (Figure 2B). ssGSEA analysis indicated that subtype I is a high PANoptosis subtype, which has a higher PANoptosis score (Figure S2E). The clinicopathological characteristics were further analyzed. Patients in subtype I were preferentially related to younger diagnosis age, higher grade, wild-type IDH, non-codeleted 1p/19q and unmethylated MGMT promoter (Figure 2C).

A total of 1,022 DEGs between two PANoptosis subtypes were identified (Figure S3A, B, Table S7). Functional enrichment analyses (GO and KEGG) indicated the enrichment of immunology and tumorigenesis related pathways in the DEGs between PANoptosis subtypes, such as interferon-gamma production and extrinsic apoptotic signaling pathway (Figure S3C, D and Table S8). Similarly, results of GSVA analysis also showed that PANoptosis subtype I was positively correlated with immune responses and tumorigenesis, whereas PANoptosis subtype II were positively associated with WNT signaling pathway (Figure 2C and Table S9). These results indicated a key role of PANoptosis in regulating tumour immune microenvironment. We next explored the status of tumor microenvironment in these two subtypes by ESTIMATE algorithm. The immune, stromal, and ESTIMATE scores of subtypes I were significantly higher, demonstrating high abundances of immune and stromal cells and low tumor purity (Figure S3E).

The infiltration abundances of 22 TIICs in each glioma sample were also compared between these two subtypes. Among them, 10 types of immune cells were obviously higher in the subtype I (macrophages, regulatory T cells, Neutrophils), whereas 7 types had lower infiltration in this subtype (activated NK cells, activated dendritic cells, activated mast cells) (Figure 2D). Furthermore, subtype I also showed a relatively higher expression level of immune checkpoints (Figure 2E), suggesting distinct TME characteristics between PANoptosis subtypes.

### Identification of PANoptosis signature in glioma from scRNA-seq Analysis

After performing quality control, normalization, and data scaling on public glioma scRNA-seq dataset GSE173278, we received a total of 26299 cells and 21530 genes for subsequent analysis (Figure S4A). These cells were clustered into 26 clusters with unsupervised Uniform Manifold Approximation and Projection (UMAP, Figure S4B). We carried out cluster annotation by R package SingleR and manual cell type annotation with cell-type-specific markers (Figure S4C). The identity of endothelial cells, fibroblasts, macrophages, oligodendrocytes, T cells and tumor cells were recognized (Figure 2F). In addition, the tumor cells were also identified based on chromosomal copy number variations with inferCNV R package (Figure S4D). Then, the PANoptosis score of each cell was obtained using the “PercentageFeatureSet” function. The cells were divided into low- and high-PANoptosis groups by their median PANoptosis score (Figure 2G). Finally, differential expression analysis was conducted between high-PANoptosis and low-PANoptosis groups. KEGG and GSEA analyses were performed with DEGs between high- and low-PANoptosis-score cells, indicating that high-PANoptosis-score cells were enriched for genes implicated in Antigen processing and presentation pathway, Cell adhesion pathway and apoptosis pathway (Figure S4E-J).

### Construction and validation of the prognostic PANRG score model

**Figure 3:**
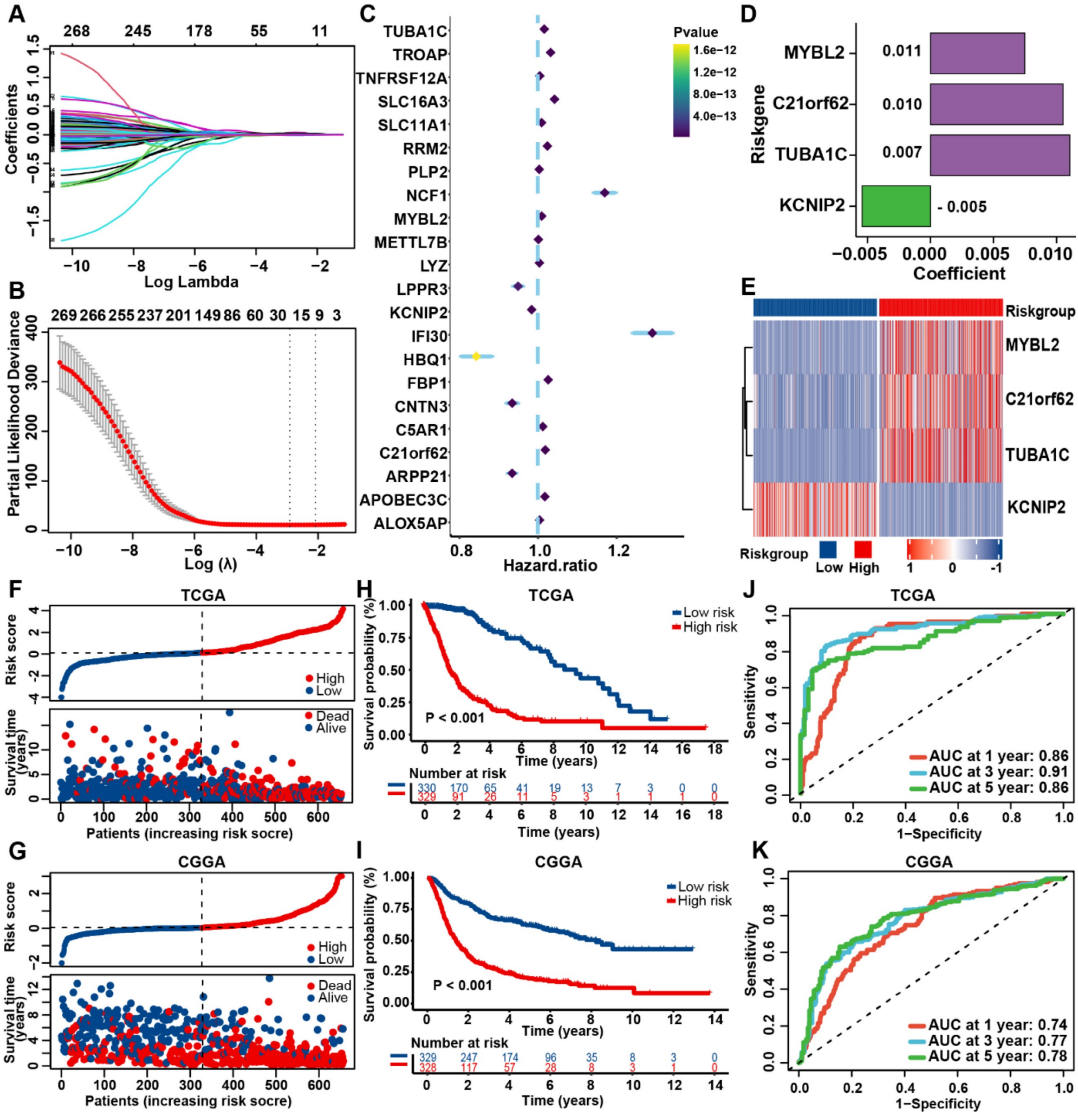
Construction and validation of the prognostic PANRG score model (A) LASSO coefficient profiles of 271 prognostic DEGs among PANoptosis subtypes. The coefficient profile plot was developed against the log (Lambda) sequence. (B) Cross-validation for turning parameter selection via minimum criteria in LASSO regression model. Two dotted vertical lines were plotted at the optimal values using the minimum criteria. (C) Forest plot of the expression profiles of 22 genes in univariate cox analysis. (D) LASSO coefficients of the 4 PANoptosis-associated predictors selected by multivariate Cox regression analysis. (E) Heatmap showing the expression levels of the 4 predictors in TCGA dataset. (F) Distribution plots of the risk score and survival status in glioma patients from the TCGA and (G) CGGA datasets. (H) The K-M curves for OS of glioma patients from TCGA and (I) CGGA datasets. (J) The ROC curve of the prognostic PANRG score model in predicting 1-, 3-, and 5-year OS in glioma patients from the TCGA and (K) CGGA datasets.

To establish a prognostic PANRG score model for prediction of OS outcome of glioma patients, 271 hub genes from bulk-seq and scRNA-seq analysis, were identified to be associated with prognosis by univariate Cox regression analysis (Table S10). By performing LASSO-Cox regression analysis in TCGA dataset, 4 PANRGs were finally identified as PANoptosis-associated predictors and applied to construct PANRG score model (Figure 3A-D, Table S11). MYBL2, C21orf62, and TUBA1C were high-risky genes and KCNIP2 was low-risk. These four genes were differentially expressed between tumor and normal tissues (Figure S5A). The expression status of these four genes in TCGA dataset are shown in Figure 3E.

Based on the median value of the prognostic PANRG score, 659 glioma patients from TCGA database were divided into low- and high-risk group. The PCA analysis demonstrated a discernible separation between two risk groups (Figure S5B, C). Patients of high-risk group have a higher PANoptosis score in TCGA and CGGA datasets (Figure S5D, E). The correlation analysis showed that MYBL2, C21orf62, TUBA1C and KCNIP2 were significantly associated with the expression of most genes that are critical for PANoptosis (Fig S5F). These 4 genes may be involved in the process of PANoptosis. The distribution plot showed that the risk score was positively correlated with glioma-death ratio of the patients (Figure 3F). In addition, the KM survival curves revealed that low-risk group had a significantly favorable OS (Figure 3H). The AUC curve was 0.86 for 1-year, 0.91 for 3-year, and 0.86 for 5-year survival, which illustrated a satisfactory prediction performance of PANRG score (Figure 3J). The results of parallel analyses performed in CGGA were quite consistent with dataset, which further confirmed the robustness of our model (Figure 3G, I, K, Figure S5G, H).

Moreover, we compared the predictive efficacy of our model with the commonly used clinical biomarkers with ROC curves 55. Results showed that our model performed better than other biomarkers for predicting the prognosis of glioma patients (Figure S5I). These findings indicated that the prognostic PANRG score model could accurately and stably predict the OS outcome of glioma patients.

### Establishment and Evaluation of a nomogram to predict survival

**Figure 4:**
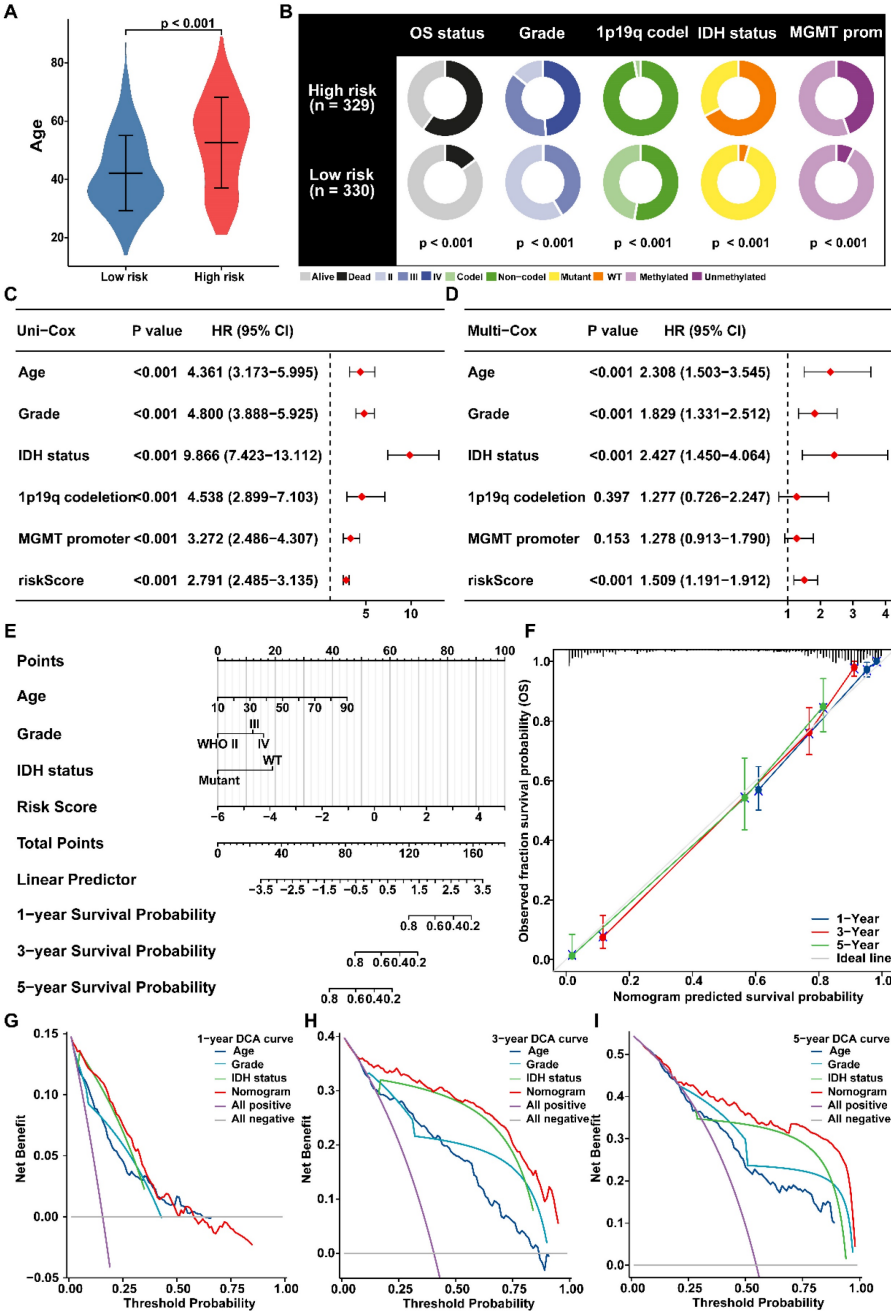
Predictive nomogram of PANRG score model (A) Comparison of the age between risk groups. (B) The distribution of patients with different clinicopathological characteristics in risk groups. (C) Univariate and (D) multivariate Cox analyses of clinical characteristics and risk score with the OS in glioma patients from TCGA. (E) Nomogram for prediction of the 1-, 3-, and 5-year OS based on risk-score, age, Grade and IDH status. (F) Calibration curves of the nomogram for prediction of 1-, 3-, and 5-year OS. (G-I) DCA curves of 3-year, 5-year, and 10-year OS for nomogram and other clinicopathological characteristics.

The correlations between PANRG score and clinical features in glioma patients were further investigated in risk groups 55. Moreover, the PANRG score was also correlated to advanced age, worse OS status, higher glioma grade and MGMT promoter unmethylated (Figure 4A, B; Figure S6A, B). Cox regression analyses further clarified that PANRG-score is always an independent prognostic factor for glioma (Figure 4C, D and Figure S6C, D).

Subsequently, we established a nomogram with independent prognostic factors to predict OS probability (Figure 4E). The accuracy of the nomogram was verified by generating the calibration and ROC curve (Figure 4F, Figure S6E), which demonstrated a satisfactory evaluation for sensitivity and specificity of OS. Besides, DCA curves illustrated that the nomogram brought more net benefit of OS than other clinical parameters (Figure 4G-I), which indicating that nomogram had better clinical utility for survival prediction in glioma patients.

### Potential Response to Chemotherapy based on the PANRG score

**Figure 5:**
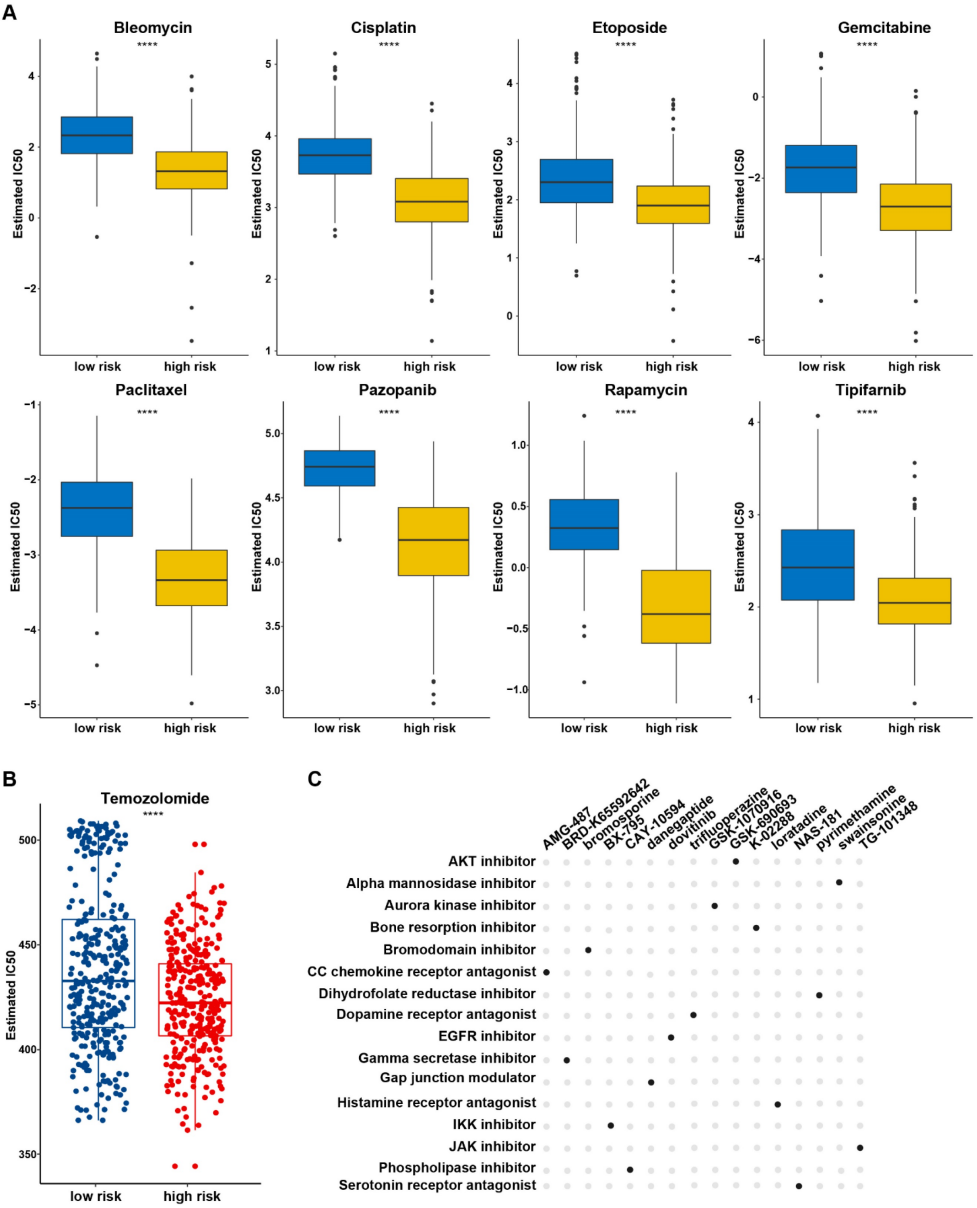
Potential compounds for glioma treatment based on the PANRG score (A) The estimated IC50 of 8 drugs with predictive high sensitivity in high-risk group based on GDSC database. (B) The estimated IC50 value of TMZ based on GDSC database between low/high risk groups. (C) Potential compounds and their MoAs shared in TCGA and CGGA datasets were identified by CMap analysis. * P < 0.05, ** P < 0.01, *** P < 0.001

Chemotherapy remains as one of the main treatment modalities for glioma and drug resistance is the primary cause of treatment failure. The relationship of the chemotherapy drug responses and PANRG-score in glioma patients was investigated based on GDSC database. The estimated IC50 of 98 drugs showed differences between two risk group (Table S12, Figure 5A), indicating that patients in high-risk group tended to benefit more from chemotherapy treatment (Figure 5B). CMap analysis was performed to explore the potential compounds and their MOA (mode of compounds action). Finally, 16 compounds shared in both datasets were identified, which provide a reference to explore potential drugs and develop personalized chemotherapy programs according to the prognostic PANRG score in glioma (Figure 5C).

### Distinct TME Characteristics in PANoptosis-related risk groups

Functional enrichment analyses were performed to characterize the biological functions of the DEGs between different PANoptosis-related risk groups. With a threshold of adj-Pvalue < 0.05 and |logFC| >2, DEGs between the two risk groups were identified (Figure S7A, B and Table S13, S14). Subsequently, GO and KEGG analyses revealed significant enrichment of these DEGs in immune and tumorigenesis-related pathways, which indicating the crucial role of PANoptosis in the regulation of TME in glioma (Figure S7C-F).

The genomic alterations in PANoptosis-related risk groups were further explored. Each risk group possessed specific top mutated genes (Figure S8A, B). IDH1 (89%) and TP53 (43%) were the most frequently mutated gene in low and high-risk group, separately. CNV analysis illustrated that patients in high-risk group tended to bear a greater CNV burden (Figure S8C). Besides, patients in high-risk group also had significantly lower MSI, which may affect the efficacy of immunotherapy (Figure S8D).

**Figure 6:**
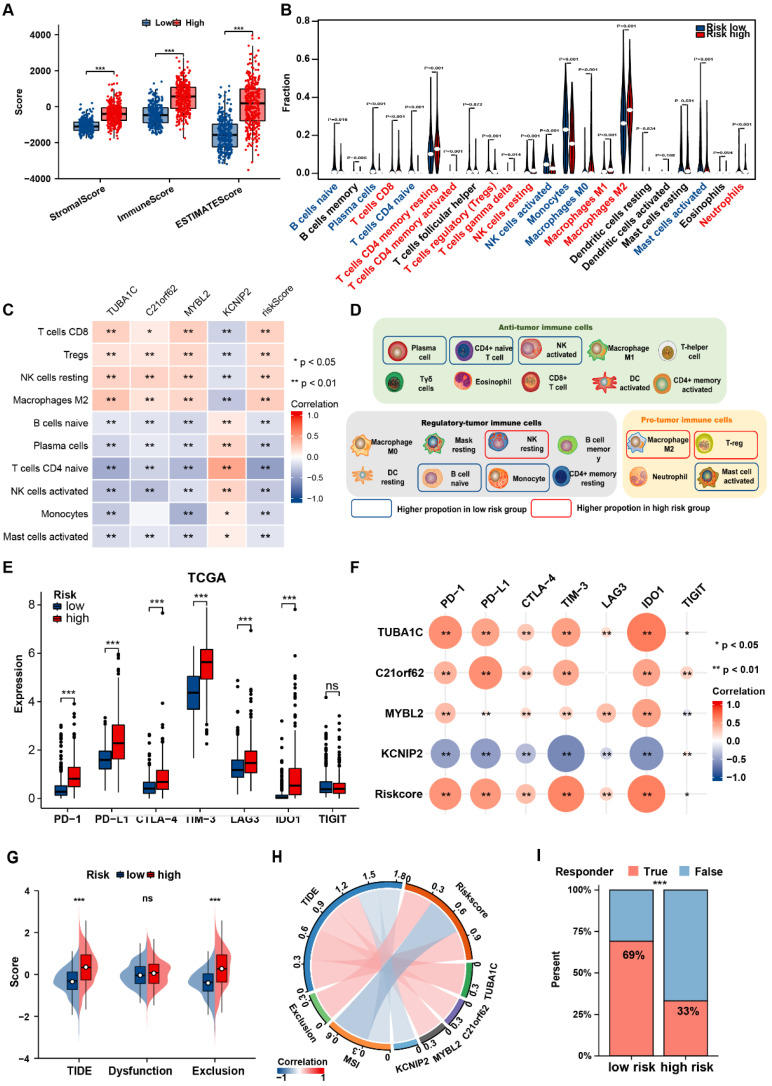
Distinct immune landscape between two PANoptosis-related risk groups (A) Boxplots showed the stromal, immune and ESTIMATE score of patients in PANoptosis-related risk groups from TCGA dataset. (B) Violin plots of the abundance of 22 types of immune cells in different risk groups in TCGA dataset. (C) The correlation between risk-score and abundance of TIICs in TCGA dataset. The color indicates the correlation coefficient. The asterisks indicate a statistically significant p-value calculated using spearman correlation analysis. (D) Schematic plot demonstrated the differences in immune cell infiltration between risk groups. The solid box indicates the cell types with consistent alteration in both TCGA and CGGA datasets. (E) Comparisons of the expression levels of 7 classical immune checkpoints between two risk groups in TCGA dataset. (F) Correlation analysis between immune checkpoints and risk score. The color and size of the circles indicate Spearman correlation coefficient. (G) Comparisons of the TIDE and exclusion scores between two risk groups. (H) The correlation between risk-score, risk gene expression levels, MSI and TIDE score. (I) Stacked histogram showed different proportions of responders and non-responders to immunotherapy between two risk groups. * P < 0.05, ** P < 0.01, *** P < 0.001

Considering the enrichment of PANoptosis-related DEGs in immunological pathways, we further investigated the correlation of the prognostic PANRG score with the TME in glioma. The high-risk group showed significantly higher immune, stroma, ESTIMATE scores (Figure 6A, Figure S9A). CIBERSORT algorithm were adopted to calculate the abundance of TIICs between the risk groups. In TCGA dataset, most pro-tumor immune cells (M2 macrophages, Tregs and neutrophils) were more abundant in high-risk group, while several types of anti-tumor immune cells (plasma cells, CD4+ naive cells, activated NK cells) had a higher infiltration in low-risk group (Figure 6B), which was consistent with the results in CGGA dataset (Figure S9B). In addition, various types of immune cells correlated with the PANRG scores and PANoptosis-associated predictors were identified in TCGA and CGGA datasets (Figure 6C, Figure S9C). Nine critical immune cells were shared by both two datasets (Figure S9D). To assist with the understanding of the TME characteristics in distinct risk groups, we summarized our results in a balance chart (Figure 6D). In general, PANoptosis-related high-risk group had a higher proportion of tumor-promoting immune cells (M2 macrophages and Tregs) and a lower proportion of antitumor immune cells (plasma cells, CD4+ naive cells and activated NK cells).

We further analyzed the associations between immune checkpoints and PANRG score. Most of the classical immune checkpoints (PD-1, PD-L1 and LAG-3, etc.) were up-regulated in high-risk group (Figure 6E, Figure S9E). PANRG score and the expression levels of PANoptosis-associated predictors were positively correlated with the expression of immune checkpoints (Figure 6F, Figure S9F). These findings indicated that the high-risk group possessed a pro-tumor microenvironment.

Finally, TIDE algorithm was applied to predict the immunotherapy responses in glioma patients. The TIDE and exclusion score were positively correlated with PANRG-risk score (Figure 6G, H and Figure S9G, H). Similarly, low-risk group had a higher proportion of responders to immunotherapy, suggesting that patients in this group may possess higher sensitivity to immunotherapy (Figure 6I and Figure S9I).

### The cell-specific expression of PANoptosis-associated predictors in glioma

**Figure 7:**
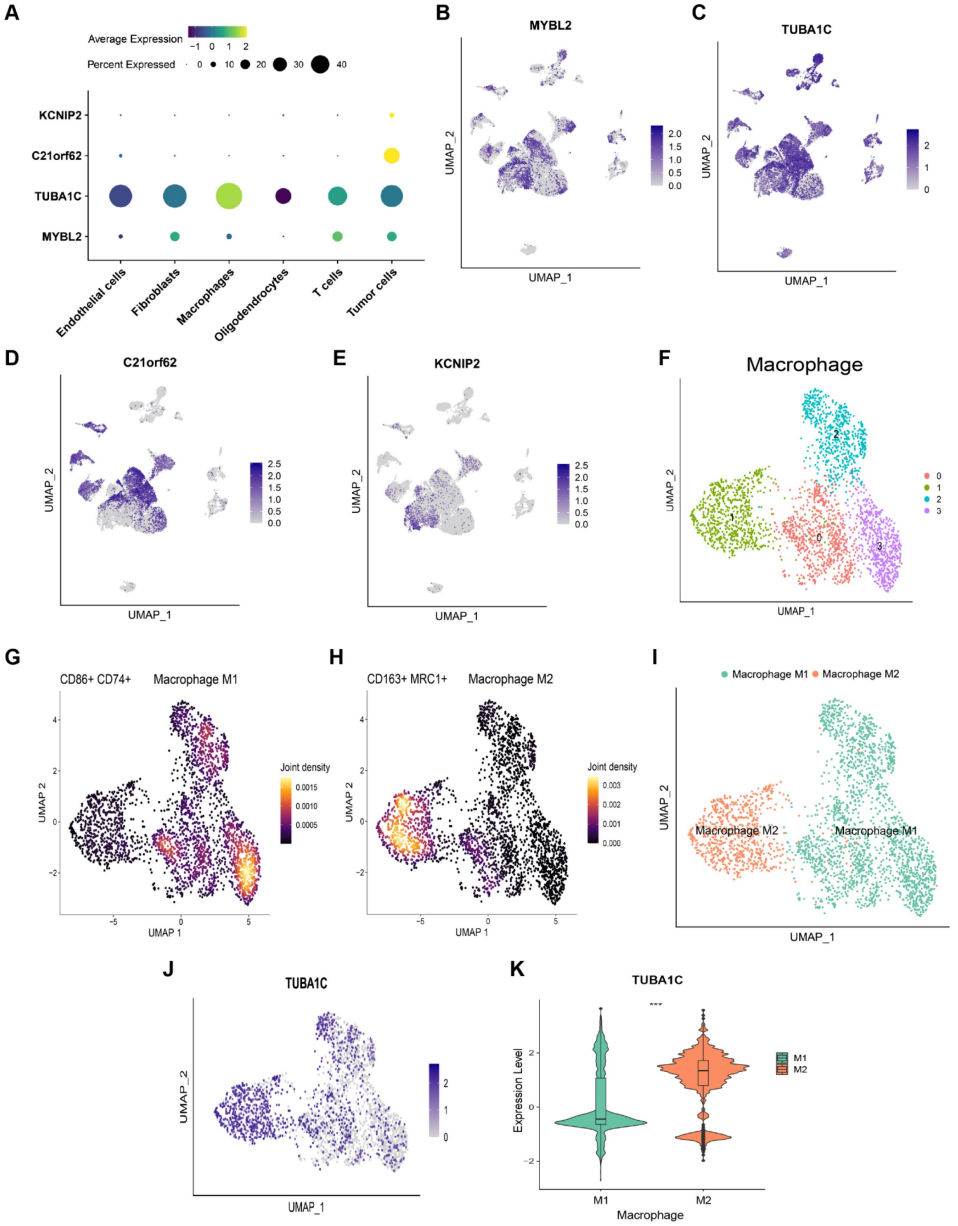
scRNA-seq analysis to explore the cell localization of 4 PANoptosis-associated predictors (A) Dot plot of the expression of 4 PANoptosis-associated predictors in different cell types. (B-E) Cell localization of MYBL2, TUBA1C, C21orf62 and KCNIP2. (F) Cluster analysis of Macrophages with UMAP. The cells were clustered into 4 clusters. (G) Density plot of the expression of M1 Macrophage markers in different clusters. (H) Density plot of the expression of M2 Macrophage markers in different clusters. (I) Annotation of Macrophage subtypes. (J) The expression of TUBA1C in M1/M2 Macrophages. (K) Comparison of the expression of TUBA1C between M1/M2 Macrophages.

The expression levels of 4 PANoptosis-associated predictors in different cell types were explored by scRNA-seq data analysis. As shown in Figure 7A-E, C21orf62 and KCNIP2 were mainly expressed in tumor cells. MYBL2 was mainly expressed in tumor cells and glioma-associated fibroblasts. KEGG functional enrichment analysis showed that genes in MYBL2-highly-expressed fibroblasts are highly enriched in cell cycle, DNA replication pathways (Figure S10A). Results of GSEA analysis revealed that MYBL2 may promote the proliferation of glioma-associated fibroblasts (Figure S10B, C). TUBA1C was mainly expressed in tumor cells and glioma-associated macrophages. Consistent with the results of Figure 6E, F, TUBA1C had a higher expression in M2 macrophages, which is crucial for tumor progression (Figure 7F-K). Thus, these predictors may play important roles in the immune microenvironment of glioma.

### Validation of the Expression Levels of PANoptosis-associated predictors in Clinical samples and Databases

**Figure 8:**
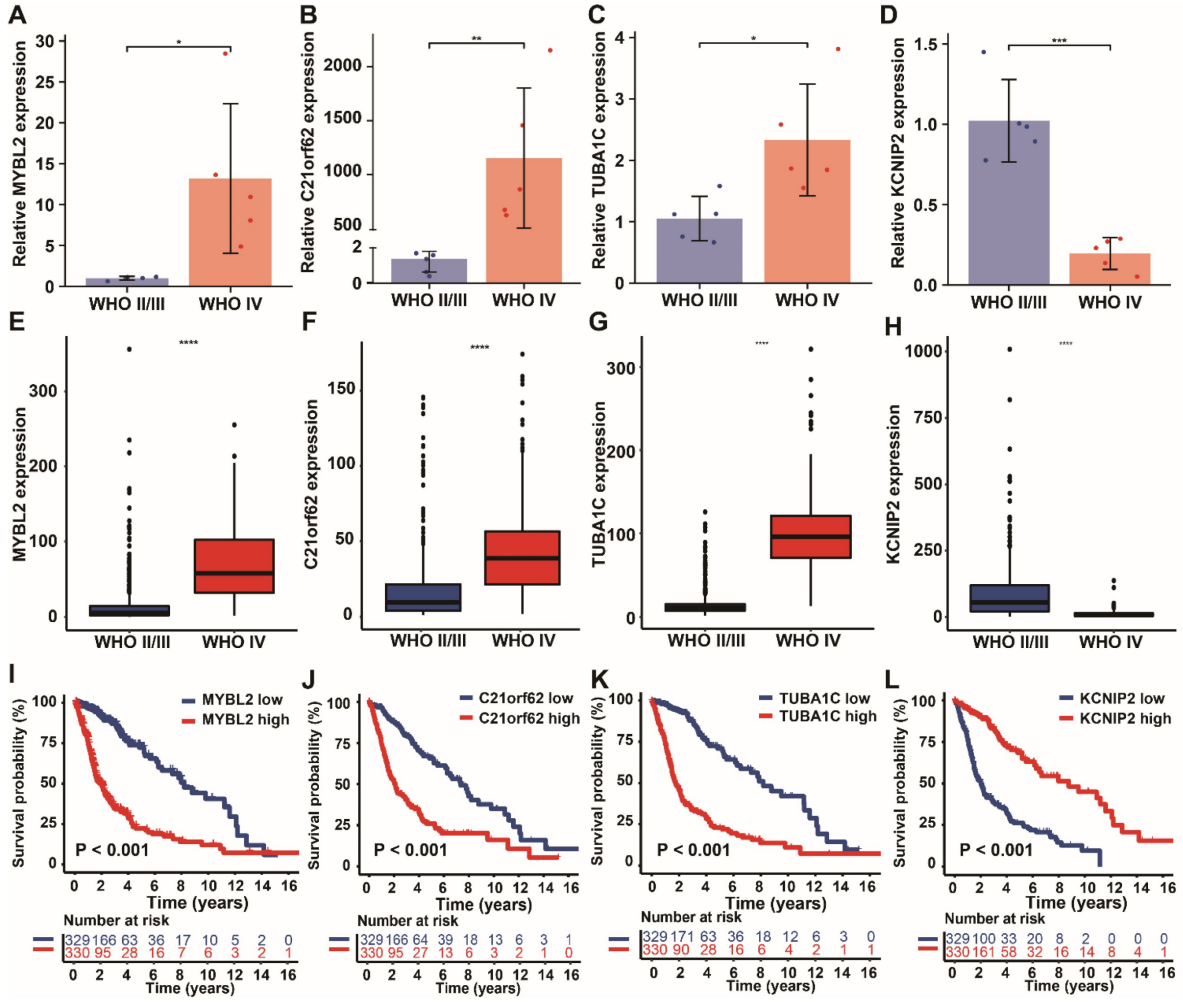
Validation of the expression levels of 4 PANoptosis-associated predictors in clinical samples. (A-D) mRNA expression levels of the 4 risk genes (A) MYBL2, (B) C21orf62, (C) TUBA1C, (D) KCNIP2 in 10 glioma human tissue samples (5 WHO II/III grade and 5 WHO IV grade glioma patient samples). (E-H) Expression analysis of (E) MYBL2, (F) C21orf62, (G) TUBA1C, (H) KCNIP2 in glioma samples with different WHO grade from TCGA dataset. (I-L) Kaplan-Meier curves of these 4 genes in glioma patients (TCGA dataset) for overall survival. * P < 0.05, ** P < 0.01, *** P < 0.001

We further validated the expression levels of the four PANoptosis-associated predictors (MYBL2, C21orf62, TUBA1C, KCNIP2) in clinical samples and databases. By using RT-qPCR assay, we detected transcriptional expression levels of these four genes in 10 glioma tissues with different malignant grades. Among them, high-risk genes MYBL2, C21orf62 and TUBA1C showed higher expression levels in tissues from high-grade gliomas patients (WHO IV grade, GBM), while the expression of low-risk gene KCNIP2 was relatively lower in GBM (Figure 8A-D), which is consistent with our previous hypothesis. Then, the expressions of these genes were also examined by transcriptome data from TCGA database (Figure 8E-H). The results further confirmed the variation tendency of these genes observed in RT-qPCR assay. Finally, the prognostic values of these genes in glioma were analyzed respectively. Kaplan-Meier curves demonstrated that low expression level of MYBL2, C21orf62, TUBA1C and high expression level of KCNIP2 were significantly associated with longer overall survival (Figure 8I-L).

### PANoptosis-associated predictors promoted proliferation and migration in glioma cells

**Figure 9:**
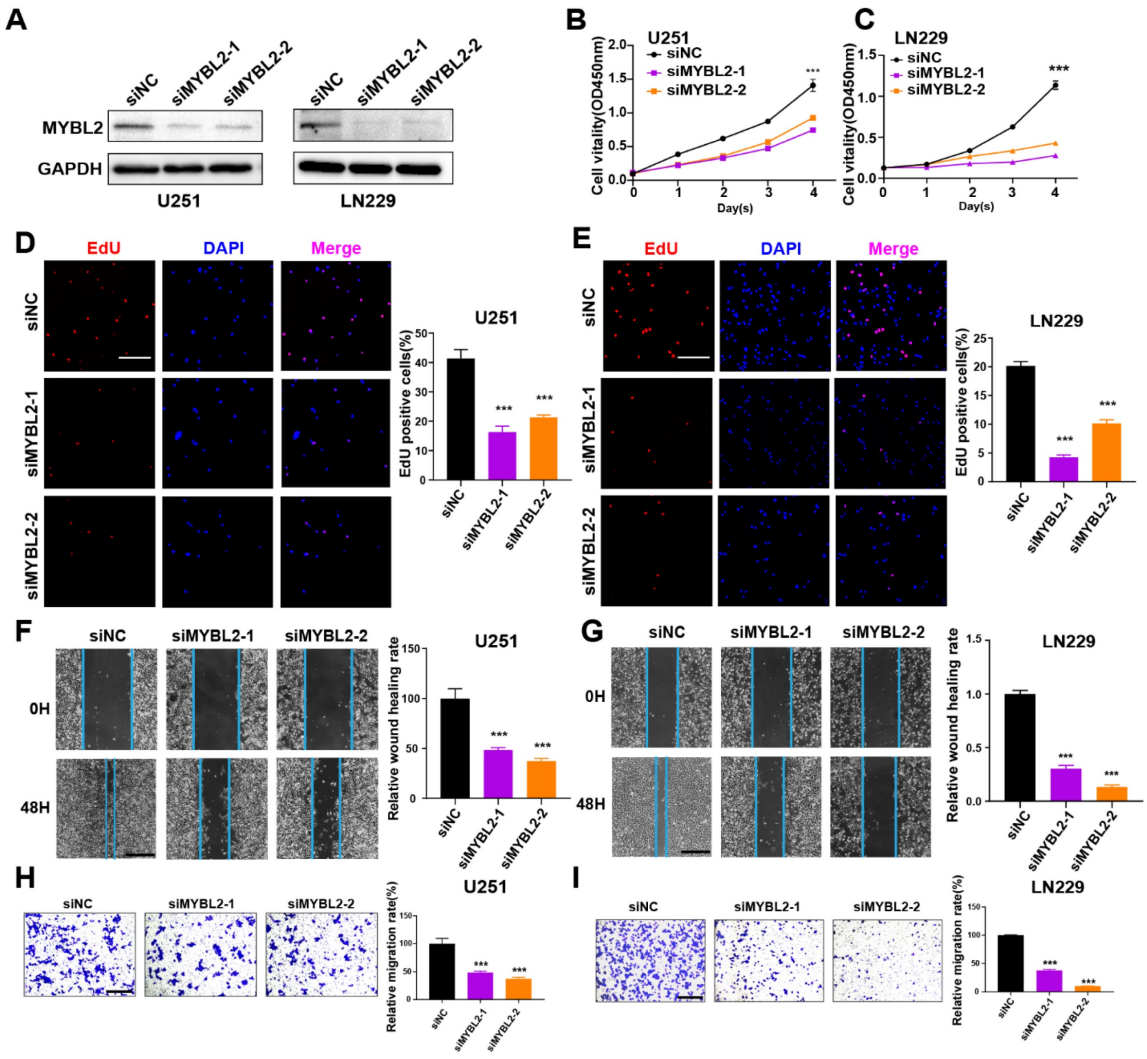
MYBL2 promotes glioma cell proliferation and migration (A) The knockdown efficiency of MYBL2 were detected by western-blot in U251 and LN229 cell line. Original blots were shown in Figure S12A, B. (B-C) CCK8 assays in U251 and LN229 cell transfected with scrambled siRNA, MYBL2 siRNAs. (D-E) EdU assays in U251 and LN229 cell transfected with scrambled siRNA, MYBL2 siRNAs. Scale bar, 100 µm. (F-G) Wound healing assays and Transwell assays in U251 and LN229 cell line transfected with scrambled siRNA, MYBL2 siRNAs. Scale bar, 500 µm. (H-I) Transwell assays in U251 and LN229 cell line transfected with scrambled siRNA, MYBL2 siRNAs. Scale bar, 500 µm; * P < 0.05, ** P < 0.01, *** P < 0.001

**Figure 10:**
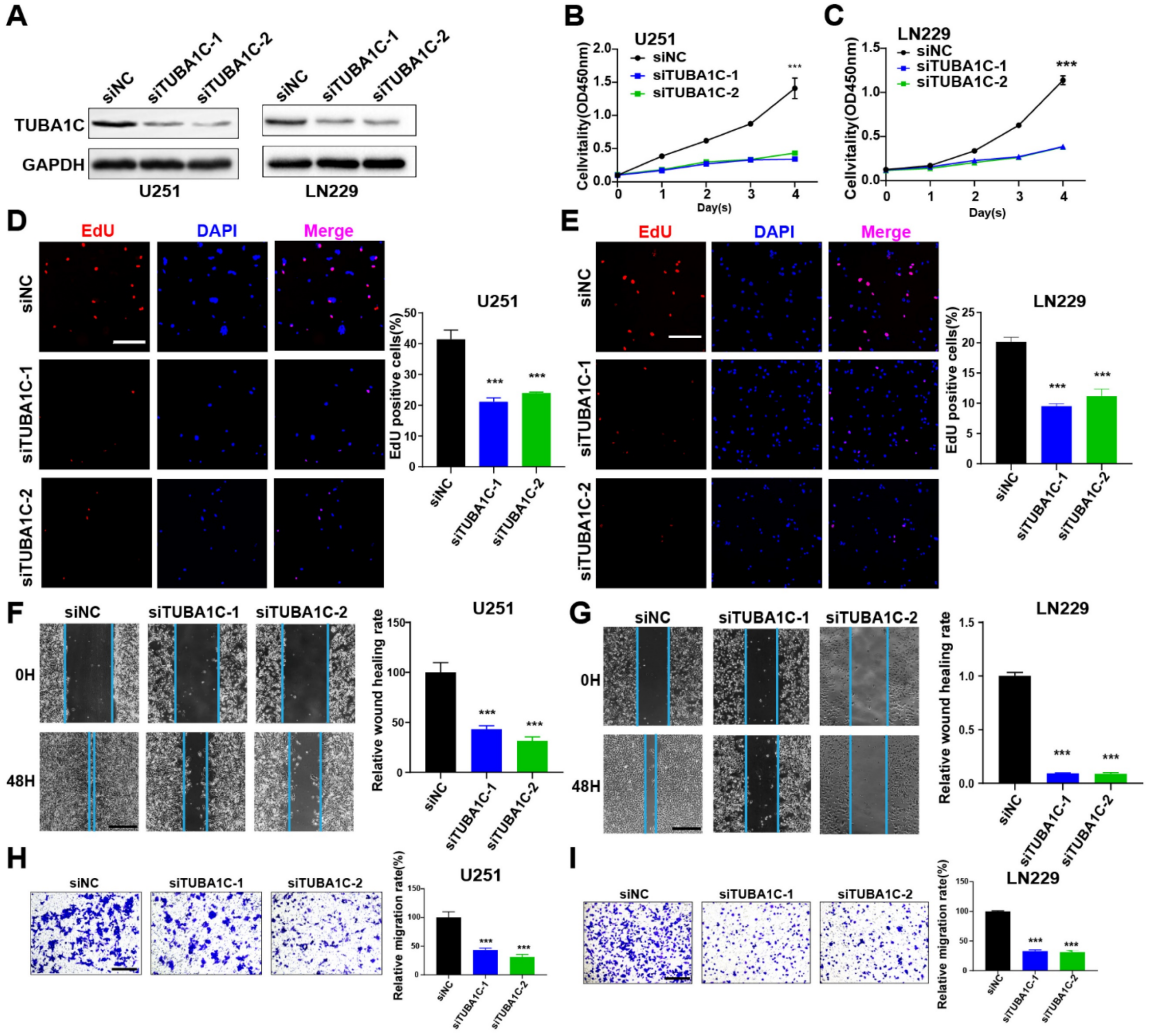
TUBA1C promotes glioma cell proliferation and migration (A) The knockdown efficiency of TUBA1C were detected by western-blot in U251 and LN229 cell line. Original blots were shown in Figure S12C, D. (B-C) CCK8 assays in U251 and LN229 cell transfected with scrambled siRNA, TUBA1C siRNAs. (D-E) EdU assays in U251 and LN229 cell transfected with scrambled siRNA, TUBA1C siRNAs. Scale bar, 100 µm (F-G) Wound healing assays and Transwell assays in U251 and LN229 cell line transfected with scrambled siRNA, TUBA1C siRNAs. Scale bar, 500 µm. (H-I) Transwell assays in U251 and LN229 cell line transfected with scrambled siRNA, TUBA1C siRNAs. Scale bar, 500 µm; * P < 0.05, ** P < 0.01, *** P < 0.001

The roles of MYBL2 and TUBA1C on tumor progression were investigated in glioma cells. These two genes were silenced by siRNAs in U251 and LN229 cells, separately. The efficacy of gene knockdown was detected by western blot and RT-qPCR (Figure 9A, 10A and Figure S11A, B). The results of cell biology assays demonstrated that knockdown of MYBL2 or TUBA1C markedly inhibited the proliferation (Figure 9B-E, Figure 10B-E); and migration of glioma cells (Figure 9F-I, Figure 10F-I). Overall, MYBL2 and TUBA1C promoted proliferation and migration, which may play an important role on glioma progression.

## Discussion

One of the most challenging barriers to establish effective treatments for glioma is the tumor heterogeneity, which encompasses a vast spectrum of molecular, genetic, microenvironment characteristics and leading to diverse therapeutic responses for different individuals 56. PCD is one of the most widely discussed subject in cancer therapy 57. Early studies of cell death usually focused on the individual PCD form in cancer. Recent studies highlight crosstalk and co-ordinations among them 58, and has led to the integration of pyroptosis, apoptosis, and necroptosis into a newly described term: PANoptosis 15. However, the regulatory mechanism and function of PANoptosis in glioma were still poorly understood. Our study revealed that the genetic landscape of PANRGs were significantly altered in glioma samples, and the PANoptosis subtypes and PANRG-scores were especially correlated with the TME of glioma patients. These results not only highlight the prognostic value of PANRGs in gliomas, but also provide clues for the understanding of the regulatory mechanisms of PANoptosis pathway in glioma.

Genetic analysis of somatic mutations showed a generally high mutation frequency of PANRGs in glioma patients. The genes with frequent genetic aberrations in glioma 59, were also reported to be closely related with the PANoptosis-associated PCD pathways, such as IDH1 60, TP53 61, 62, EGFR, ATRX 63. Additionally, the most frequently mutated genes varied in different PANoptosis-related risk groups, suggesting that the mutations of those genes may not only influence the prognosis of glioma, but also lead to different PANoptosis status. Besides, the prognostic PANRG score model we established here, displayed a better prognostic prediction efficacy than those commonly used clinical biomarkers.

Given that PANoptosis is an inflammatory PCD cascade which could be observed during infections and autoinflammatory diseases 64, it may also play an important role in immunoregulation during the progression of glioma. Consistent with this hypothesis, DEGs between both PANoptosis subtypes and risk groups were highly enriched in immunological pathways. The major cellular component of TME, the TIICs, including granulocytes, lymphocytes, and macrophages, which are closely related to the progression and therapeutic response of tumors 65. The effects of PANRGs on tumor immune landscape were investigated in glioma here. The abundance of 22 TIICs were statistically different between the PANoptosis subtypes and risk groups. Consistent with previous studies, the infiltration of immunosuppressive TIICs, such as Tregs 66 and macrophages M2 67, which usually favor the tumor growth by suppressing the anti-cancer immune response, were highly enriched in subtype I and the high-risk group with worse OS. The consequences of different immune cells in the TME were not always consistent between different analyses, since the physiological function of these cells were based on the type, anatomical site and the stage of the tumor. Generally, these results indicated a strong connection between the PANRGs and TME of glioma.

Distinct tumor microenvironment and immunogenomic patterns of PANoptosis-related risk groups suggested different sensitivity to immunotherapy. Here, we observed a higher expression of classical immune checkpoints in the high-risk group. Nevertheless, results of TIDE demonstrated patients in the low-risk group were more likely to respond to immunotherapy. The effectiveness of ICB therapy could be affected by multiple factors, including the cytotoxic T cell infiltration. For the T cell exclusion modelling, cancer-associated fibroblasts, myeloid-derived suppressor cells and the M2 tumor-associated macrophages are usually examined 68. The highly enrichment of immunosuppressive cells in the high-risk group, such as macrophages M2 may restrict the activity of cytotoxic T cells, leading to a poor outcome for ICB therapy. Furthermore, considering the distinct potential drug effect spectrums of patients in different risk-groups, the PANRG-score may also provide guidance in the selection of personalized medicines for glioma patients.

MYBL2, a member of the MYB transcription factor family, has been implicated in the pathogenesis of various malignancies. In breast cancer, MYBL2 overexpression is associated with aggressive tumor phenotypes and poorer prognosis 69, 70. Its oncogenic potential is linked to its ability to regulate cell cycle progression, proliferation, and apoptosis evasion. Similarly, in colorectal cancer 71 and lung cancer 72, MYBL2 has been found to promote tumorigenicity. C21orf62 is a gene with not fully understood functions. KCNIP2, a potassium channel-interacting protein, regulates membrane excitability and ion channel function. In GBM, KCNIP2 was downregulated in tumor tissues and appears to be significantly linked to the overall survival of patients 73. TUBA1C, a tubulin alpha family protein, is crucial for maintaining cytoskeletal integrity. Aberrant expression of TUBA1C has been reported in several malignancies, including pancreatic ductal adenocarcinoma 74, breast cancer 75 and bladder urothelial carcinoma 76. Its dysregulation can lead to cytoskeletal defects that facilitate tumor cell proliferation, migration, and invasion. In this study, we found that MYBL2 and TUBA1C, highly expressed in high-grade gliomas, may be involved in the process of PANoptosis and promoted the proliferation and migration of glioma cells. MYBL2 may promote the proliferation of glioma-associated fibroblasts and TUBA1C had a high expression in M2 macrophages, which confirms and expands on the results of previous studies.

Overall, our study revealed the prognostic value of PANRGs and provided evidence for the involvement of PANoptosis in the progression and tumor microenvironment of glioma. Nevertheless, the mechanism of PANoptosis has not been fully elucidated. Thus, identification and validation of the function of various PANRGs in glioma will be an important direction for our future research.

## Conclusions

Our study identified two PANoptosis subtypes in glioma patients. Four PANoptosis-related predictors (MYBL2, TUBA1C, C21orf62 and KCNIP2) were identified and predictive PANRG score model was developed for glioma based on bulk-seq and scRNA-seq analysis, which is significantly associated with tumor immune landscape and therapeutic responses. The expression and function of PANoptosis-associated predictors was explored in clinical samples and glioma cells. These findings highlighted the crucial clinical implications of PANRGs in the prognosis and individualized treatment of glioma patients.

## Supplementary Material

Supplementary figures and tables.
