# Supplementary material for: Identification of PANoptosis-related predictors for prognosis and tumor microenvironment by multiomics analysis in glioma
Source: J Cancer. 2024 Mar 11;15(9):2486–504. doi: 10.7150/jca.94200 (PMC10988298; doi:10.7150/jca.94200)
Supplement: Supplementary file 1 — Supplementary figures and tables. [file jcav15p2486s1.pdf]

Supplementary Figures

Figure S1

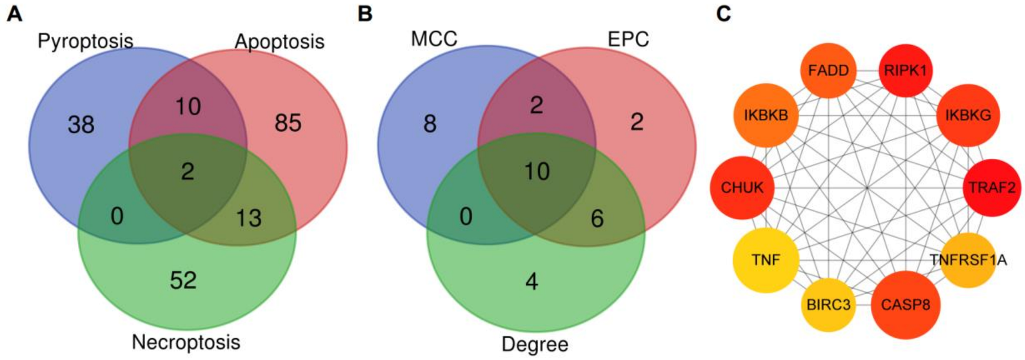

Figure S1. PPI analysis of PANRGs (A) Distribution of PANoptosis-related genes (PANRGs) among pyroptosis, apoptosis and necroptosis pathways. (B) Venn diagram of the ten critical hub genes that identified by three methods (MCC, EPC and Degree) in cytoHubba plugin. (C) The core network of protein-protein interaction of these ten hub genes.

Figure S2

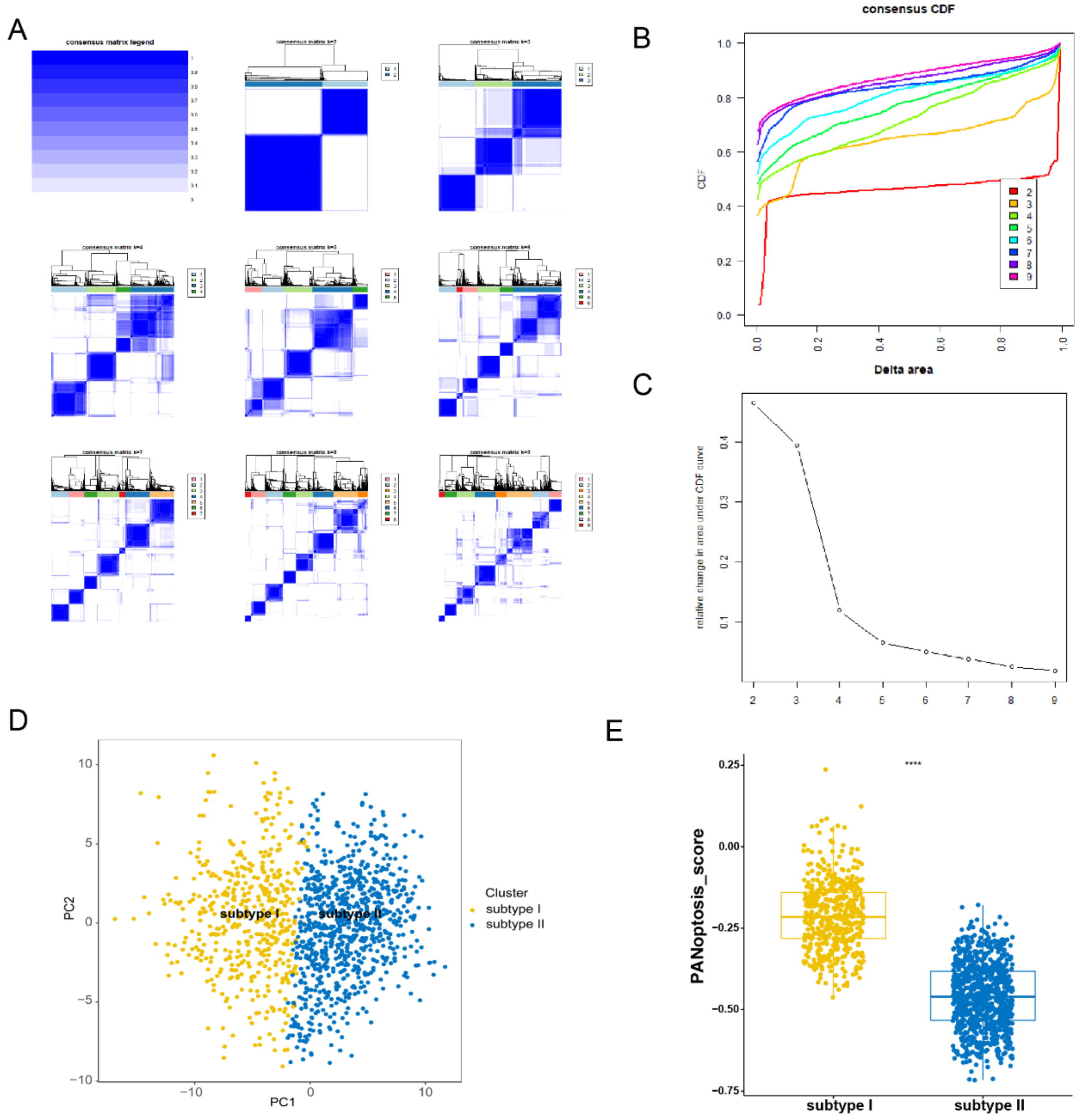

Figure S2. Unsupervised clustering with pyroptosis-related genes (A) Consensus matrix heatmaps for k = 1-9. (B) CDF curves of the consensus score from k = 2 to 9. (C) The relative change in the area under the CDF curve from k = 2 to 9. (D) PCA analysis between the two PANoptosis subtypes. (E) Comparison of PANoptosis score between PANoptosis Subtypes.

Figure S3

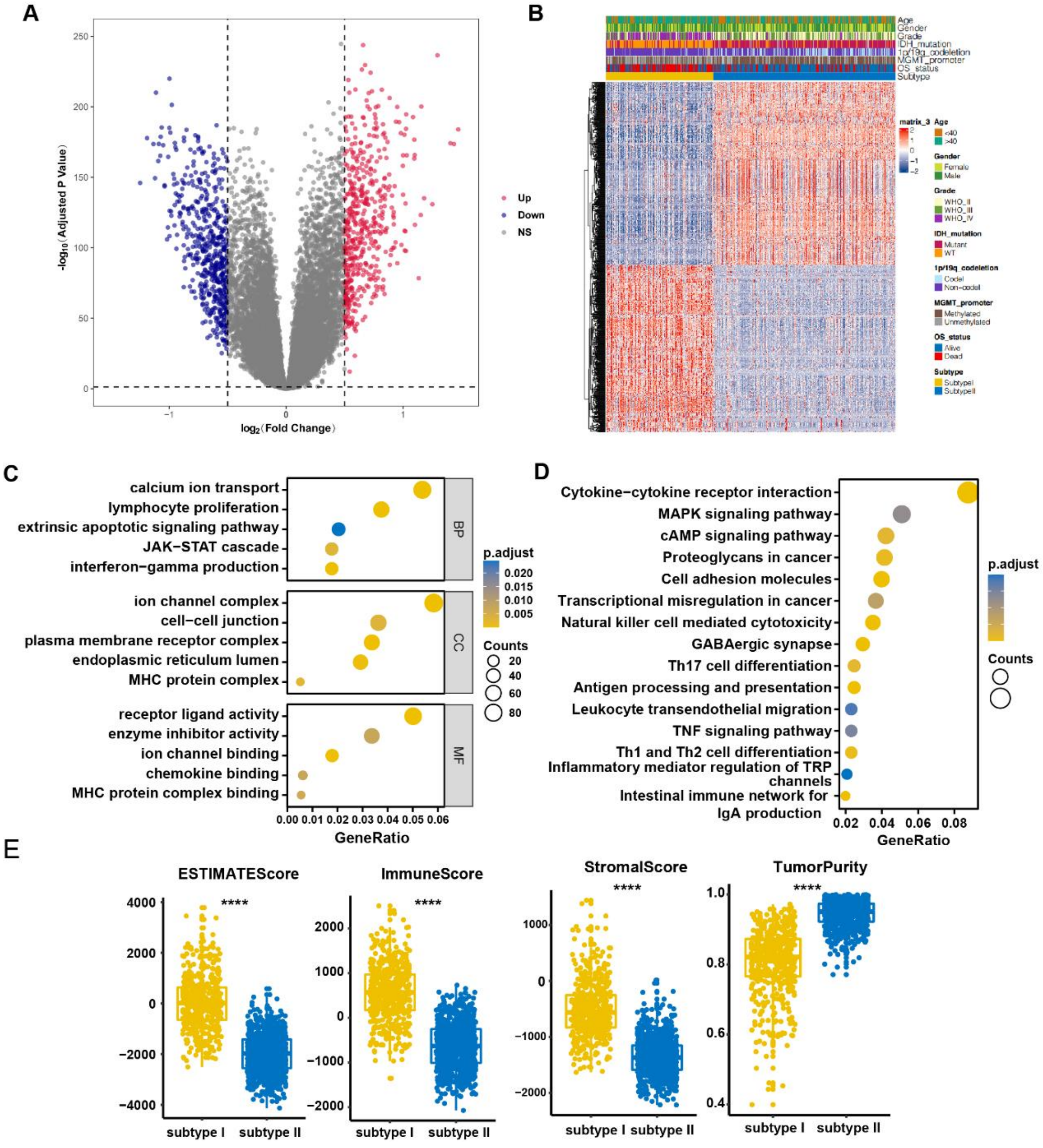

Figure S3. Differential expression analysis between the two PANoptosis subtypes (A) Volcano plot shows differentially expressed genes (DEGs) between two PANoptosis subtypes. (B) The heatmap of the DEGs between PANoptosis subtypes. (C) GO analysis. (D) KEGG analysis. (E) Comparisons of stromal score, immune score, ESTIMATE score and tumor purity between PANoptosis Subtypes.

Figure S4

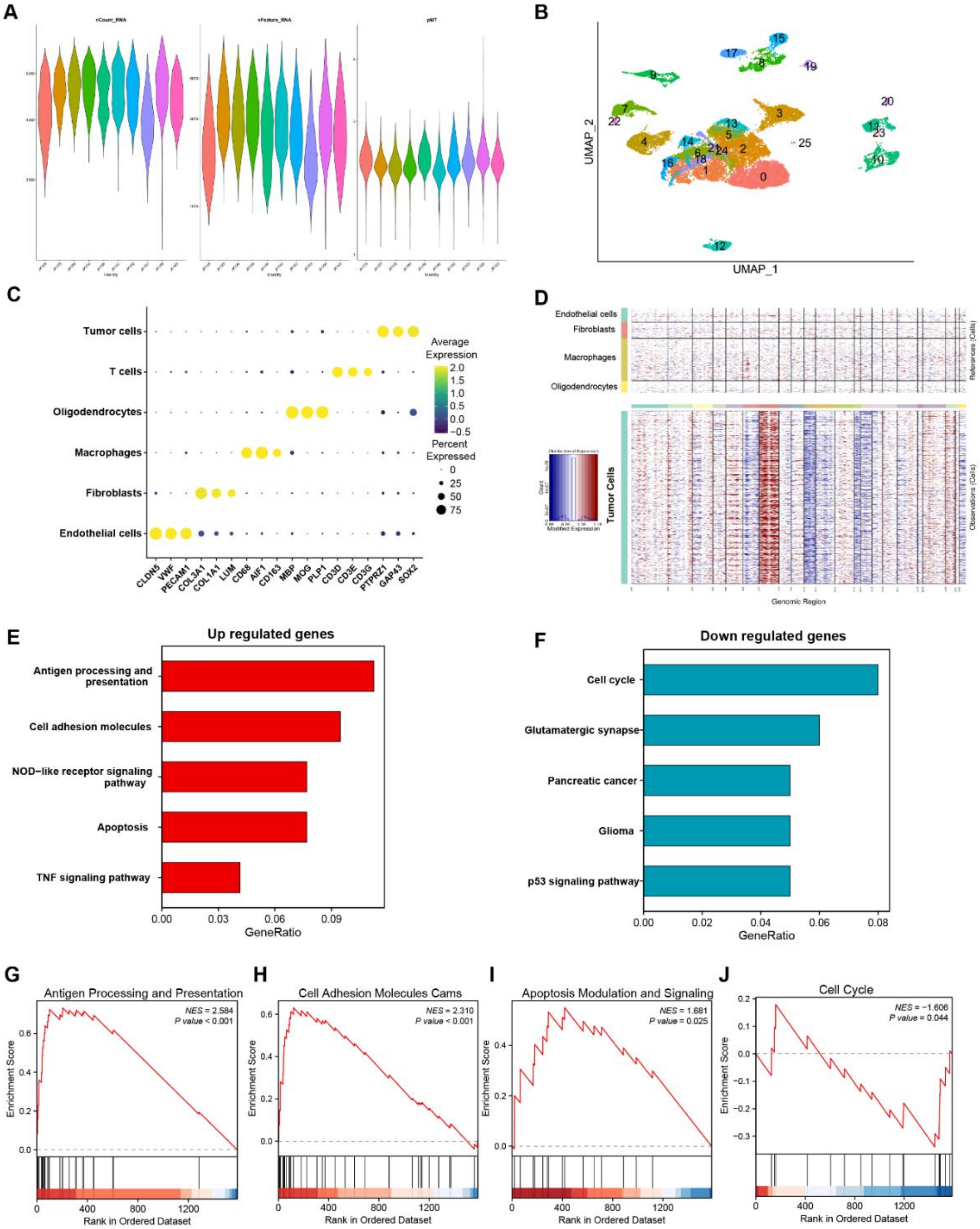

Figure S4. scRNA-seq data analysis (A) Violin plots of UMI counts, gene counts and mitochondrial ratio in each patient. (B) Cluster analysis with UMAP. The cells were clustered into 26 clusters. (C) Expression of cell-type-specific markers in different cell types. (D) The heatmap of obvious copy number variations (CNVs) in malignant tumor cells. Red representing amplifications, blue representing deletions. (E) KEGG analysis of up-regulated genes. (F) KEGG analysis of down-regulated genes. (G-J) GSEA analysis in scRNA-seq data.

Figure S5

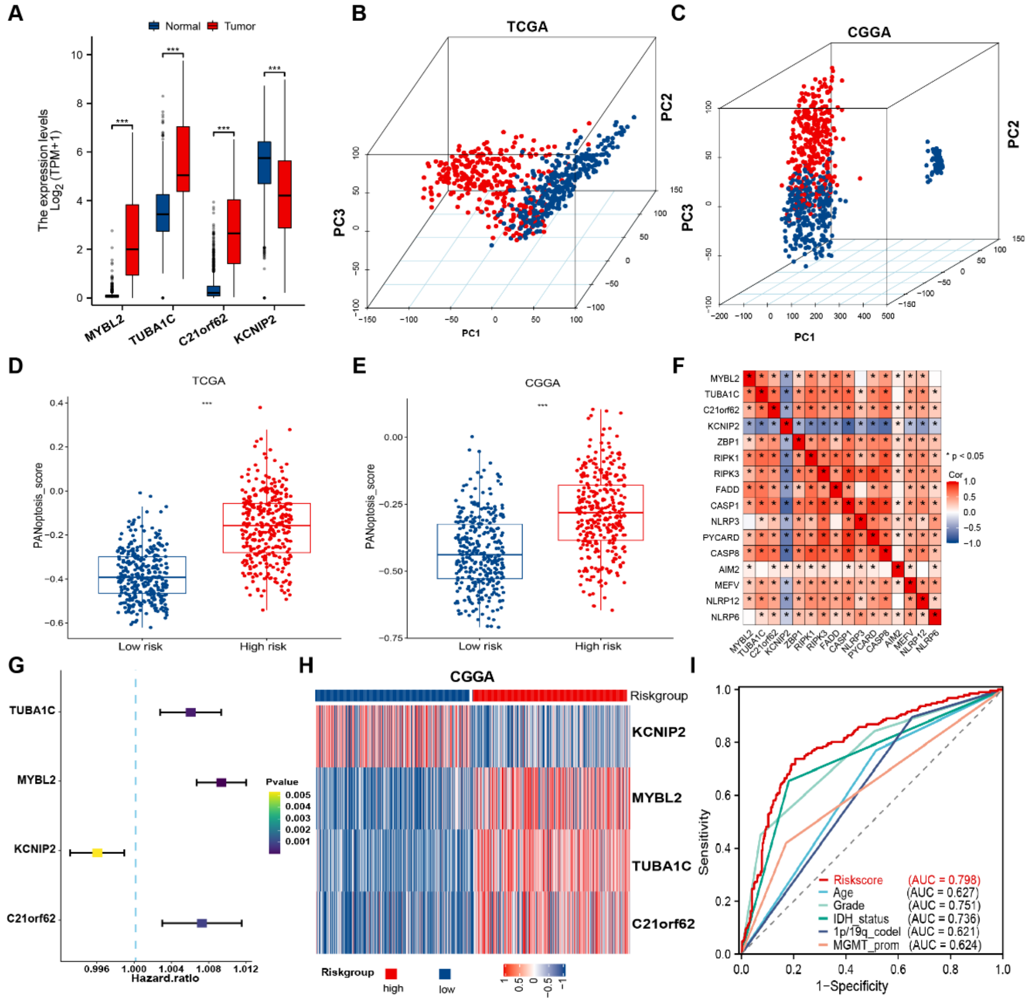

Figure S5. Construction and validation of the prognostic PANRG_score model (A) The expression levels of the 4 predictors between tumor and normal samples in TCGA dataset. (B) PCA analysis showed a remarkable difference in transcriptomes between the two risk groups both in (B)TCGA and (C) CGGA datasets. (D) PANoptosis score between high- and low-risk groups in (D)TCGA and (E)CGGA datasets. (F) The expression correlation analysis between the 4 predictors and PANoptosis marker genes. (G) Multivariate Cox regression analysis of the 4 PANoptosis-associated predictors in CGGA datasets. (H) Heatmap showing the expression levels of the 4 predictors in CGGA dataset. (I) ROC curves about risk score, Age, Grade, IDH status, 1p/19q codeletion and MGMT promoter status in TCGA dataset.

Figure S6

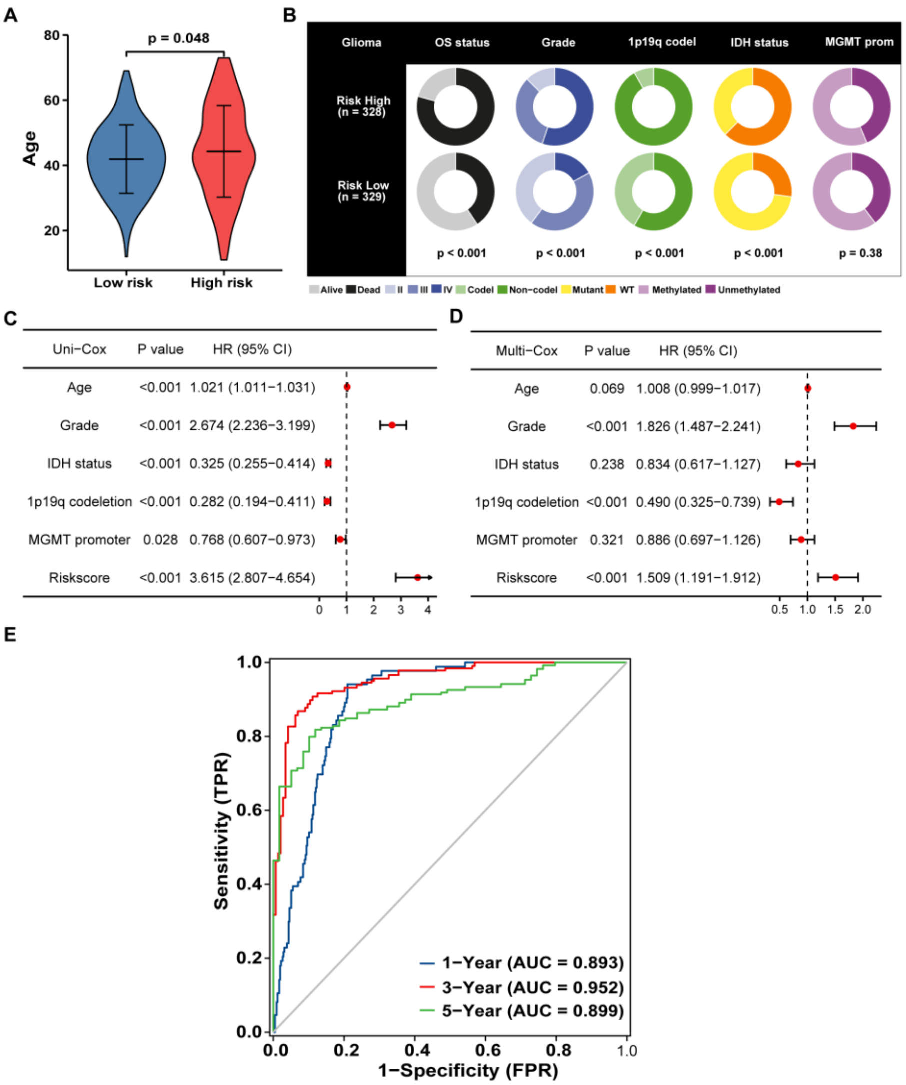

Figure S6. Correlation Analysis Between the prognostic PANRG_score and clinicopathological characteristics in CGGA dataset (A)Comparison of the age of glioma patients between the high/low-risk groups in CGGA dataset. (B) The distribution of patients with different clinicopathological characteristics in the high/low-risk groups in CGGA dataset. (C) Univariate and (D) multivariate Cox analyses of the clinical characteristics and risk score with the overall survival (OS) in CGGA dataset. (E) Time-dependent ROC curves of the nomogram for 1-,3-, and 5-year survivals in CGGA dataset.

Figure S7

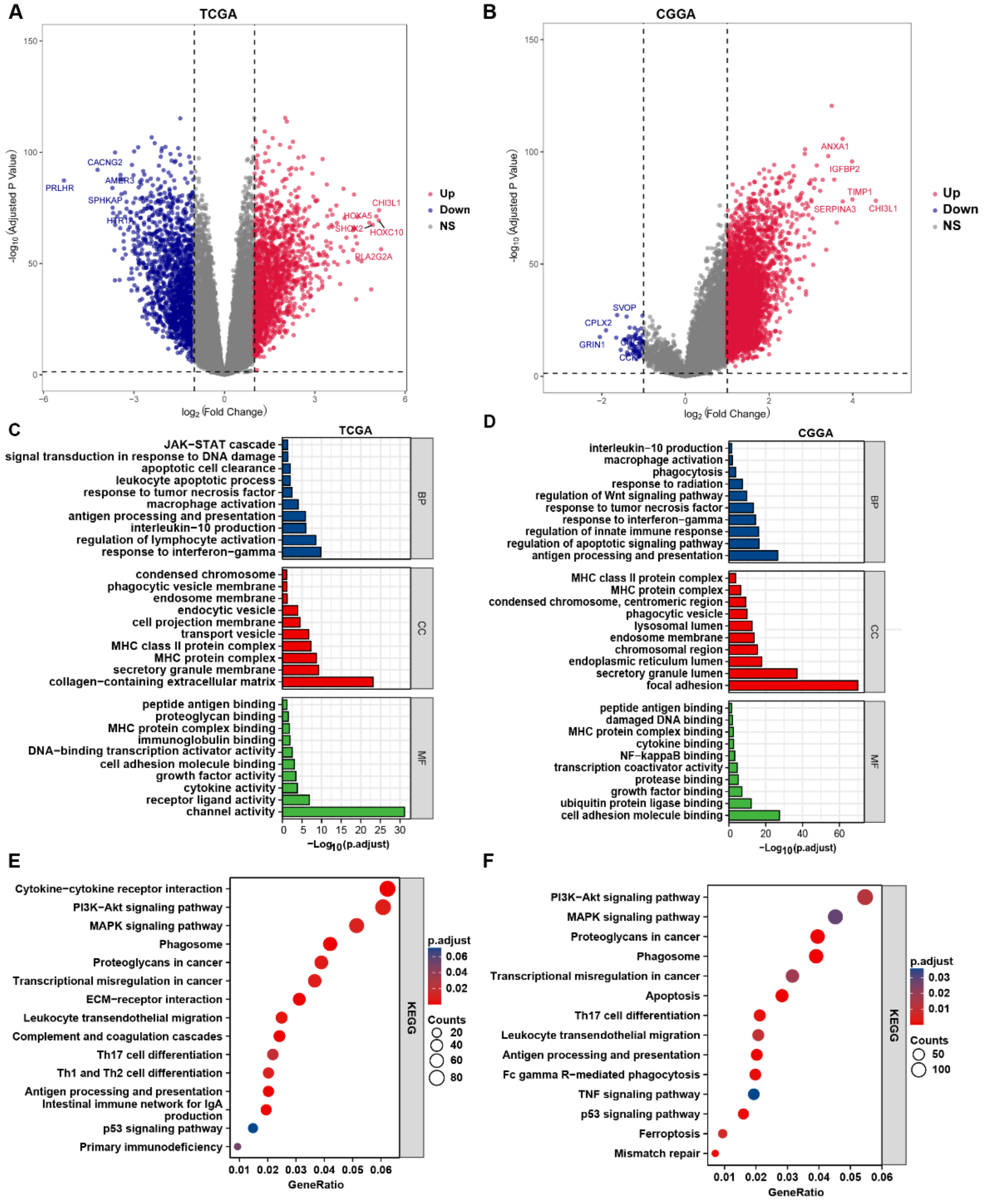

Figure S7. Differential expression and GO/KEGG enrichment analysis between PANoptosis-related risk groups in TCGA and CGGA datasets (A) Volcano plot of DEGs between PANoptosis-related risk groups in TCGA and (B) CGGA datasets. (C) GO enrichment analysis based on DEGs between the two risk groups in TCGA and (D) CGGA datasets. (E) KEGG analysis of DEGs between different PANoptosis-related risk groups in the TCGA and (F) CGGA datasets.

Figure S8

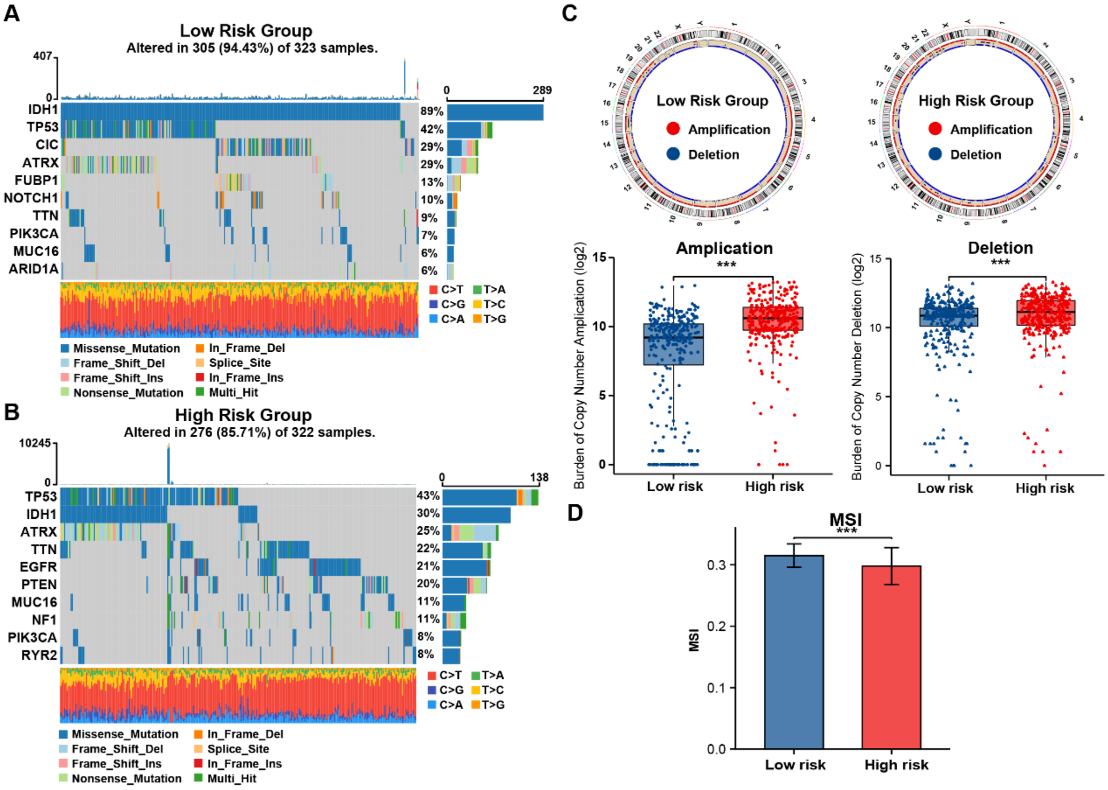

Figure S8. Genomic variations of two PANoptosis-related risk groups (A) Top 10 most frequently mutated genes in low and (B) high-risk groups. (C) The amplifications and deletions of genes in each PANoptosis -related risk group in corresponding chromosomes. Red dots representing amplifications, blue dots representing deletions, black dots representing no significant CNVs (Up panel). Boxplots showed burdens of copy number amplifications and deletions in these two groups (Down panel). (D) Microsatellite Instability (MSI) of the low/high-risk groups. * $P < 0.05$, ** $P < 0.01$, *** $P < 0.001$

Figure S9

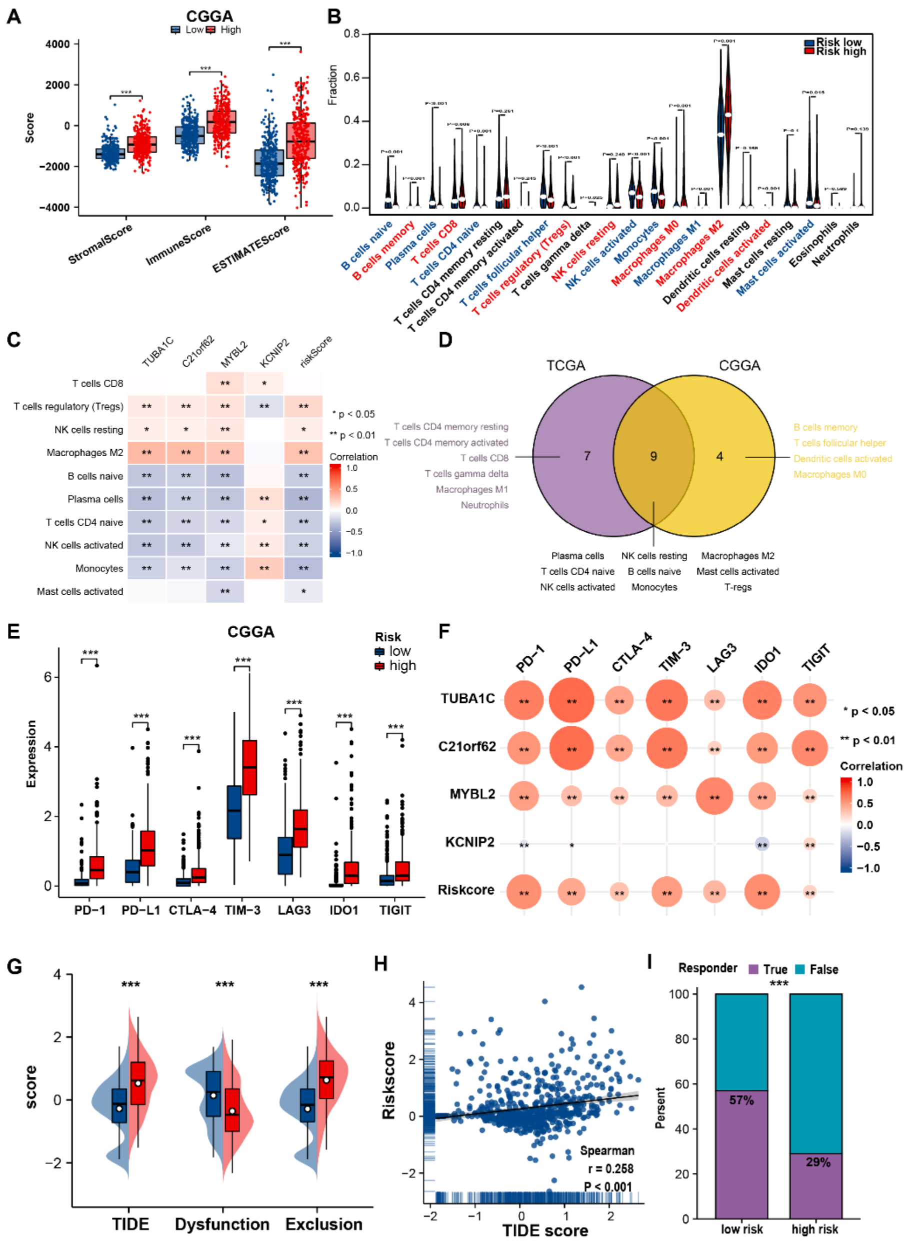

Figure S9. Distinct immune landscape between two PANoptosis-related risk groups in CGGA dataset (A) Boxplots showed the stromal, immune and ESTIMATE score of patients in PANoptosis-related risk groups. (B) The abundance of 22 types of TIICs in different risk groups in CGGA dataset. (C) The correlation between risk-score and abundance of TIICs. The color indicates the correlation coefficient. The asterisks indicate a statistically significant p-value calculated using spearman correlation analysis. (D) The intersection of critical immune cell types between TCGA and CGGA datasets. (E) Comparisons of the expression levels of 7 classical immune checkpoints between two risk groups. (F)

Correlation analysis between immune checkpoints and risk score. The color and size of the circles indicate Spearman correlation coefficient. (G) Comparisons of the TIDE and exclusion scores between two risk groups. (H) The correlation between risk-score, risk gene expression levels, MSI and TIDE score. (I) Stacked histogram showed different proportions of responders and non-responders to immunotherapy between two risk groups. * $P < 0.05$, ** $P < 0.01$, *** $P < 0.001$

Figure S10

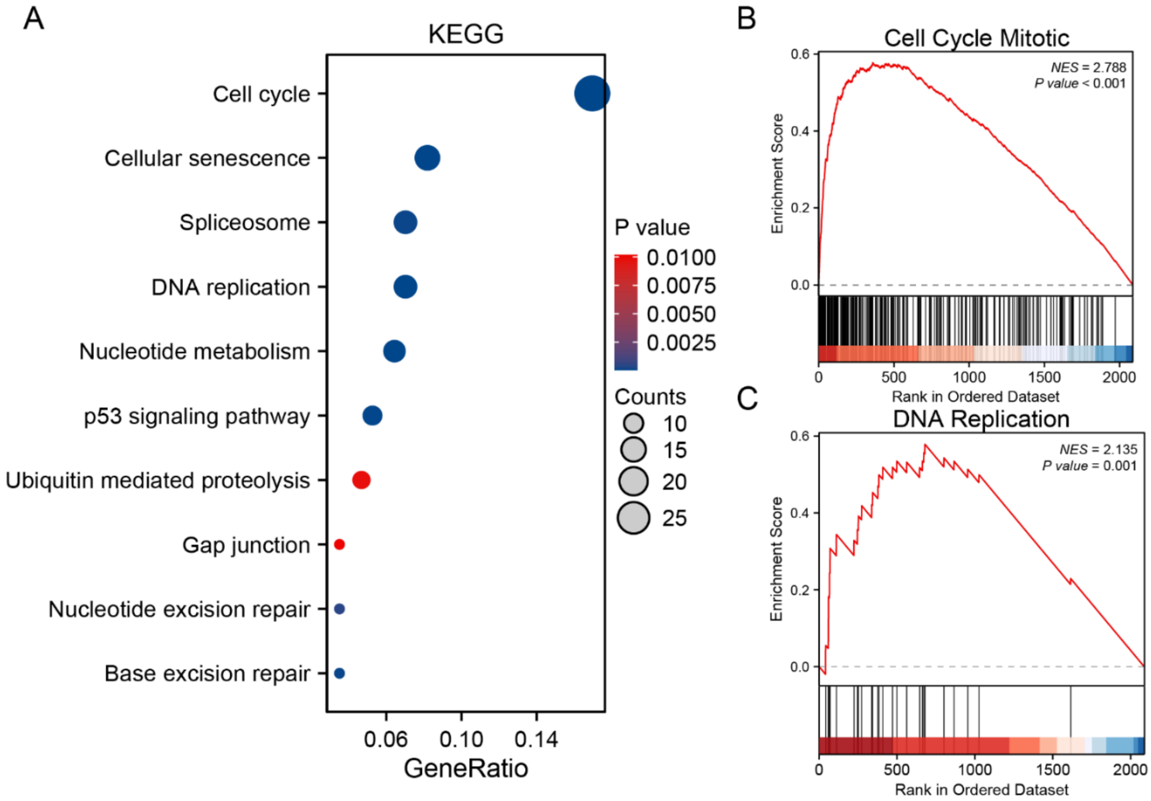

Figure S10. Functional enrichment analysis of MYBL2 in glioma-associated fibroblasts. (A) KEGG analysis of up-regulated genes in MYBL2 highly-expressed fibroblasts. (B, C) GSEA analysis of MYBL2 in glioma-associated fibroblasts.

Figure S11

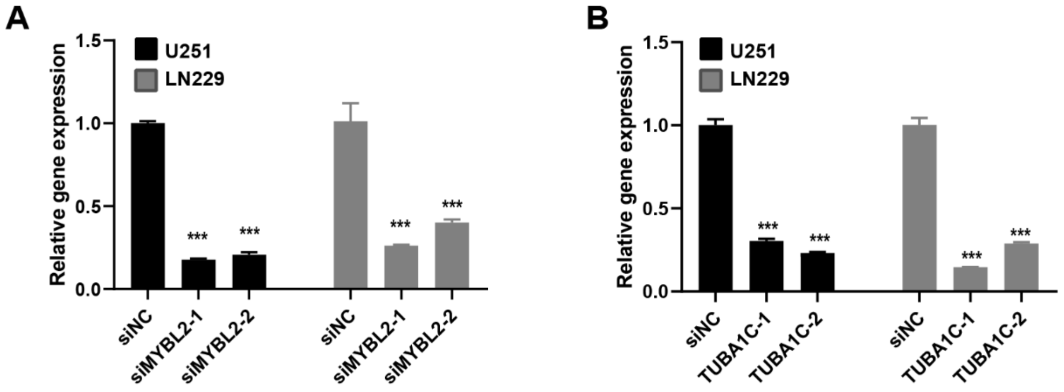

Figure S11. Knockdown of MYBL2 and TUBA1C in glioma cells (A, B) Relative expression of MYBL2 and TUBA1C in U251 and LN229 cell lines transfected with control, MYBL2 siRNAs and TUBA1C siRNAs. * $P < 0.05$, ** $P < 0.01$, *** $P < 0.001$

Figure S12

Original Images for Blots

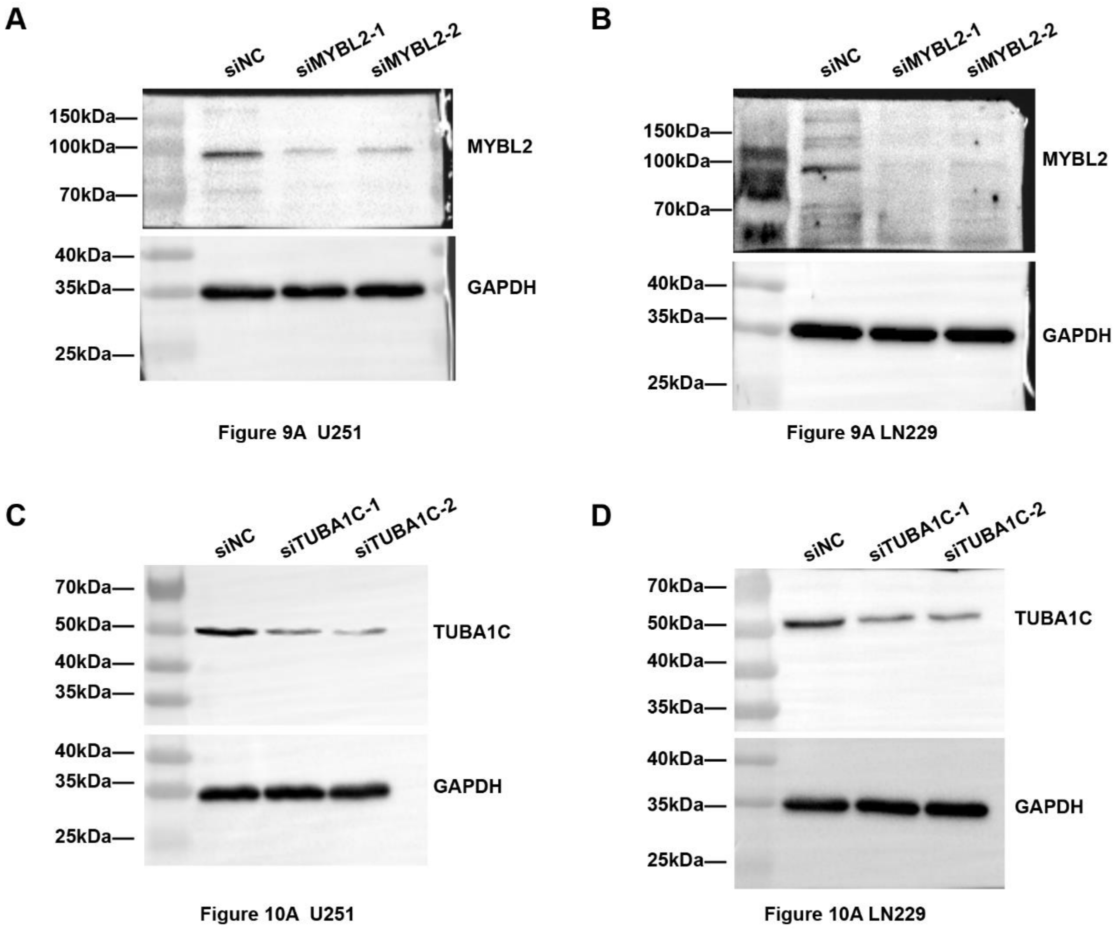

Figure S12. Original Images for Western Blots (A, B) Original Images for Western Blots of MYBL2 knockdown in U251 and LN229 cell lines. (C, D) Original Images for Western Blots of TUBA1C knockdown in U251 and LN229 cell lines.

Supplementary Tables

Table S1: General Clinical information of 1316 glioma patients

Characteristic		Datasets	
		TCGA	CGGA
Total		659	657
Age(years), median		46 (35, 59)	43 (34.75, 52)
Gender, n (%)	Female	280 (42.5%)	283 (43.1%)
	Male	379 (57.5%)	374 (56.9%)
OS tatus, n (%)	Alive	409 (62.3%)	263 (40%)
	Dead	247 (37.7%)	394 (60%)
Grade, n (%)	WHO II	214 (35.5%)	172 (26.2%)
	WHO III	236 (39.1%)	248 (37.7%)
	WHO IV	153 (25.4%)	237 (36.1%)
IDH status, n (%)	Mutant	419 (64.3%)	333 (54.7%)
	WT	233 (35.7%)	276 (45.3%)
1p19q codeletion, n (%)	Codel	165 (25.3%)	137 (23.2%)
	Non-codel	488 (74.7%)	454 (76.8%)
MGMT promoter, n (%)	Methylated	470 (74.8%)	304 (58.2%)
	Unmethylated	158 (25.2%)	218 (41.8%)

Table S2：Summary of 200 recognized PANoptosis-related genes

Type	Gene
Apoptosis	ANP32A,APAF1,AVEN,BAD,BAK1,BAX,BBC3,BCL2,BCL2A1,BCL2L1,BCL2L10,BCL2L11,BCL2L12,BCL2L14,BCL2L2,BID,BIK,BIRC2,BIRC3,BMF,BNIP3,BNIP3L,CASP10,CASP3,CASP6,CASP7,CASP8,CASP9,CFLAR,CRADD,CSE1L,CYCS,DIABLO,FADD,FAS,FASLG,HRK,MCL1,MOAP1,PMAIP1,RIPK1,SH3GLB1,TNF,TNFRSF10A,TNFRSF10B,TNFRSF10C,TNFRSF10D,TNFRSF1A,TNFRSF1B,TNFSF10,TP53,TRADD,XIAP,AIFM1,AKT1,AKT2,AKT3,ATM,CAPN1,CAPN2,CHP1,CHP2,CHUK,CSF2RB,DFFA,DFFB,ENDOD1,ENDOG,EXOG,IKBKB,IKBKG,IL1A,IL1B,IL1R1,IL1RAP,IL3,IL3RA,IRAK1,IRAK2,IRAK3,IRAK4,MAP3K14,MYD88,NFKB1,NFKBIA,NGF,NTRK1,PIK3CA,PIK3CB,PIK3CD,PIK3CG,PIK3R1,PIK3R2,PIK3R3,PIK3R5,PPP3CA,PPP3CB,PPP3CC,PPP3R1,PPP3R2,PRKACA,PRKACB,PRKACG,PRKAR1A,PRKAR1B,PRKAR2A,PRKAR2B,PRKX,RELA,TRAF2
Pyroptosis	AIM2,BAK1,BAX,CASP1,CASP3,CASP4,CASP5,CASP6,CASP8,CASP9,CHMP2A,CHMP2B,CHMP4B,CHMP4C,CHMP6,CHMP7,CYCS,ELANE,GPX4,GSDMB,GSDMC,GSDMD,GZMB,HMGB1,IL18,IL1A,IL1B,IL6,IRF1,IRF2,NLRC4,NLRP1,NLRP2,NLRP3,NLRP6,NLRP7,NOD1,NOD2,PLCG1,PRKACA,PYCARD,SCAF11,TP53,TP63,TIRAP,TNF,GZMA,GSDMA,GSDME,PJVK
Necroptosis	ZBP1,USP22,TSC1,TRIM11,TRAF2,TNFSF10,TNFRSF21,TNFRSF1B,TNFRSF1A,TNF,TLR3,TERT,TARDBP,STUB1,STAT3,SQSTM1,SPATA2,SLC39A7,SIRT3,SIRT2,SIRT1,RNF31,RIPK3,RIPK1,PLK1,PANX1,OTULIN,MYCN,MYC,MPG,MLKL,MAPK8,MAP3K7,LEF1,KLF9,ITPK1,IPMK,IDH2,IDH1,ID1,HSPA4,HSP90AA1,HDAC9,HAT1,GATA3,FLT3,FASLG,FAS,FADD,EGFR,DNMT1,DIABLO,DDX58,CYLD,CFLAR,CDKN2A,CD40,CASP8,BRAF,BNIP3,BCL2L11,BCL2,BACH2,AXL,ATRX,APP,ALK

Table S3: The siRNA sequences

Genes	Forward (5’ to 3’)	Reverse (5’ to 3’)
siMYBL2-1	CCCAGAUCAGAAGUACUCCAUTT	AUGGAGUACUUCUGAUCUGGGTT
siMYBL2-2	GCUUGGUGUGACCUGAGUAAATT	UUUACUCAGGUCACACCAAGCTT
siTUBA1C-1	CCCACAGUCAUUGAUGAAGUUTT	AACUUCAUCAAUGACUGUGGGTT
siTUBA1C-2	GCUGCCCUUGAGAAGGAUUAUTT	AUAAUCCUUCUCAAGGGCAGCTT
siNC	UUCUCCGAACGUGUCACGU	ACGUGACACGUUCGGAGAA

Table S4: Antibody for Western blotting

Reagent	Company	Identifier
B-Myb Polyclonal antibody	Proteintech, China	18896-1-AP
TUBA1C Antibody	SAB, USA	42796
GAPDH Monoclonal antibody	Proteintech, China	60004-1-Ig
HRP-conjugated Goat Anti-Rabbit IgG	Proteintech, China	SA00001-2
HRP-conjugated Goat Anti-Mouse IgG	Proteintech, China	SA00001-1

Table S5: The primer sequences for qRT-PCR

Genes	Forward (5' to 3')	Reverse (5' to 3')
C21orf62	AGAGAGTGGACGCACATACAG	TTGTCGAGTGTTAGCAAATCCG
MYBL2	ACAGATTCAGATGTGCCGGAG	TTCCAGTCCTGCTGTCCAAA
TUBA1C	TCTTCAGTGAAACGGGTGCT	TGATGAGTTGCTCAGGGTGG
KCNIP2	AATTCACGCGCAAGGAGTTG	AGGTGCTGGAGTCTCCTTGA
GAPDH	ACAACTTTGGTATCGTGGAAGG	GCCATCACGCCACAGTTTC

Table S6：Top 20 hub genes screened by EPC, MCC and Degree method

Rank	EPC	MCC	Degree
1	XIAP	BIRC3	RIPK1
2	TRAF2	FAS	IKBKG
3	TP53	RIPK3	AKT1
4	TNFRSF1A	FASLG	TRAF2
5	TNF	TRAF2	TP53
6	RIPK1	TNFRSF10B	TNFRSF1A
7	RELA	IKBKB	RELA
8	NFKBIA	TNFRSF10A	FADD
9	NFKB1	TNF	PIK3CA
10	IKBKG	IKBKG	BCL2L1
11	IKBKB	CHUK	CASP3
12	FAS	CFLAR	PIK3R1
13	FADD	TNFSF10	IL1B
14	CHUK	TRADD	TNF
15	CASP8	RIPK1	CASP8
16	CASP3	CASP8	IKBKB
17	BIRC3	CASP10	HSP90AA1
18	BIRC2	TNFRSF1A	NFKB1
19	BCL2L1	BIRC2	BIRC3
20	AKT1	FADD	CHUK

Table S7: Top 25 differential expressed genes between the two PANoptosis subtypes

symbol	logFC	adj.P.Val
APOBEC3G	0.658842104	1.80E-244
ABCC3	1.295016406	2.85E-237
CASP4	0.675965684	2.61E-230
C1RL	0.648362022	4.16E-227
PLAUR	0.722559895	6.19E-225
PAK7	-0.997303549	1.15E-220
C1R	0.532463229	9.22E-220
ANXA2	0.593225962	5.84E-213
EMP3	0.818942121	8.16E-213
CCDC109B	0.734547739	9.60E-212
SP100	0.540245211	8.50E-211
CSMD3	-1.113578995	9.11E-211
CISH	0.707346209	1.85E-210
CLCF1	0.885924945	6.04E-207
PLBD1	0.688342911	3.30E-205
SHANK2	-0.977971875	4.70E-202
MYO1G	0.787353187	2.69E-201
SERPINA1	0.696895615	5.23E-201
TREM1	1.157122215	6.88E-201
SRPX2	0.950772976	1.24E-200
STEAP3	0.767752618	2.20E-200
DENND2D	0.715511653	4.83E-199
PTPN7	0.758955041	2.06E-195
FCGR2B	1.027432996	2.22E-195
APOBEC3C	0.535260175	7.96E-194

Table S8: GO and KEGG enrichment analysis of DEGs between the two PANoptosis subtypes

ONTOLOGY	Description	GeneRatio	p.adjust
BP	calcium ion transport	79/1469	2.489E-10
BP	lymphocyte proliferation	55/1469	6.212E-09
BP	interferon-gamma production	26/1469	1.909E-05
BP	JAK-STAT cascade	26/1469	0.0028899
BP	extrinsic apoptotic signaling pathway	30/1469	0.0243355
CC	ion channel complex	90/1544	1.25E-27
CC	plasma membrane receptor complex	52/1544	3.742E-07
CC	endoplasmic reticulum lumen	45/1544	0.0002949
CC	MHC protein complex	8/1544	0.0025787
CC	cell-cell junction	56/1544	0.0033402
MF	receptor ligand activity	73/1457	6.445E-06
MF	enzyme inhibitor activity	49/1457	0.0073418
MF	ion channel binding	26/1457	0.0001341
MF	chemokine binding	9/1457	0.0073418
MF	MHC protein complex binding	8/1457	0.0062806
KEGG	Cytokine-cytokine receptor interaction	57/710	3.987E-07
KEGG	GABAergic synapse	25/710	3.534E-06
KEGG	Cell adhesion molecules	34/710	4.139E-06
KEGG	Intestinal immune network for IgA production	14/710	0.0005353
KEGG	cAMP signaling pathway	36/710	0.0010341
KEGG	Th17 cell differentiation	22/710	0.0010341
KEGG	TNF signaling pathway	22/710	0.001812
KEGG	Th1 and Th2 cell differentiation	19/710	0.0022185
KEGG	Proteoglycans in cancer	33/710	0.0028264
KEGG	MAPK signaling pathway	42/710	0.005548
KEGG	Antigen processing and presentation	16/710	0.005548
KEGG	Leukocyte transendothelial migration	19/710	0.021753
KEGG	Natural killer cell mediated cytotoxicity	21/710	0.0218804
KEGG	Transcriptional misregulation in cancer	28/710	0.0226245
KEGG	Inflammatory mediator regulation of TRP channels	17/710	0.0254981

Table S9: The activation states of biological pathways in distinct PANoptosis subtypes by GSVA enrichment analysis

term	logFC	adj.P.Val
ANTIGEN_PROCESSING_AND_PRESENTATION	0.51	3E-183
APOPTOSIS	0.33	1E-121
B_CELL_RECEPTOR_SIGNALING_PATHWAY	0.26	7.3E-61
BETA_ALANINE_METABOLISM	-0.22	3.4E-37
CALCIUM_SIGNALING_PATHWAY	-0.23	4.2E-50
COMPLEMENT_AND_COAGULATION_CASCADES	0.58	7E-258
CYTOKINE_CYTOKINE_RECEPTOR_INTERACTION	0.47	1E-245
CYTOSOLIC_DNA_SENSING_PATHWAY	0.42	5E-148
DNA_REPLICATION	0.33	7.3E-47
ECM_RECEPTOR_INTERACTION	0.43	3E-155
ERBB_SIGNALING_PATHWAY	-0.21	3.3E-45
GLUTATHIONE_METABOLISM	0.39	2E-120
GLYCOSAMINOGLYCAN_BIOSYNTHESIS_KERATAN_SULFATE	0.47	3E-114
GLYCOSAMINOGLYCAN_DEGRADATION	0.57	1E-188
INTESTINAL_IMMUNE_NETWORK_FOR_IGA_PRODUCTION	0.62	2E-223
JAK_STAT_SIGNALING_PATHWAY	0.35	2E-122
LEUKOCYTE_TRANSENDOTHELIAL_MIGRATION	0.36	2E-136
MISMATCH_REPAIR	0.37	7.7E-60
MTOR_SIGNALING_PATHWAY	-0.18	2.1E-32
NATURAL_KILLER_CELL_MEDIATED_CYTOTOXICITY	0.33	7E-125
NEUROACTIVE_LIGAND_RECEPTOR_INTERACTION	-0.23	1.6E-57
NOD_LIKE_RECEPTOR_SIGNALING_PATHWAY	0.31	5.6E-89
OTHER_GLYCAN_DEGRADATION	0.59	3E-178
P53_SIGNALING_PATHWAY	0.32	5.1E-94
PHOSPHATIDYLINOSITOL_SIGNALING_SYSTEM	-0.26	2.2E-54
PRIMARY_IMMUNODEFICIENCY	0.57	2E-205
SYSTEMIC_LUPUS_ERYTHEMATOSUS	0.62	9E-232
T_CELL_RECEPTOR_SIGNALING_PATHWAY	0.24	2.6E-62
TOLL_LIKE_RECEPTOR_SIGNALING_PATHWAY	0.39	4E-145
WNT_SIGNALING_PATHWAY	-0.28	2E-99

Table S10: Prognostic analysis of top25 DEGs identified by univariate Cox regression

id	HR	CI.95LL	CI.95HL	pvalue
GJB6	0.992344	0.986867	0.997852	0.006502
CAMK1G	0.992426	0.987374	0.997503	0.0035
NEFL	0.998655	0.997789	0.999522	0.002364
CCK	0.996484	0.994244	0.998729	0.002155
SRRM4	0.977132	0.963087	0.991382	0.001738
WNT10B	0.968994	0.950306	0.988051	0.001526
CALY	0.992501	0.988011	0.997011	0.001139
VIP	0.971539	0.954886	0.988482	0.001063
KCNJ4	0.993044	0.988905	0.997201	0.001057
SLC32A1	0.988857	0.98225	0.99551	0.001055
GPR22	0.951003	0.923244	0.979598	0.000888
SLC6A7	0.974477	0.959764	0.989415	0.000865
ANO3	0.940783	0.907878	0.974881	0.000778
VSNL1	0.998507	0.997651	0.999364	0.000646
OLFM3	0.967613	0.949512	0.986058	0.000633
NEUROD6	0.972043	0.956469	0.987871	0.00058
MAL2	0.985357	0.977171	0.993611	0.000529
SV2B	0.988948	0.982782	0.995153	0.000497
KCNV1	0.953517	0.928335	0.979382	0.000491
CCKBR	0.967554	0.949839	0.9856	0.000468
SST	0.996079	0.993898	0.998265	0.000443
RBFOX1	0.985841	0.97804	0.993704	0.000435
CAMK2A	0.998546	0.997737	0.999355	0.000429
SYT1	0.998236	0.997262	0.99921	0.000388
SLC7A4	0.92574	0.887274	0.965874	0.000366

Table S11: Prognostic analysis of 4 PANoptosis-associated predictors by multivariate Cox regression

id	HR	HR.95L	HR.95H	pvalue
MYBL2	1.009076	1.006456	1.011703	9.64E-12
TUBA1C	1.005824	1.002595	1.009064	4.01E-04
C21orf62	1.007008	1.002812	1.011223	0.001047
KCNIP2	0.995919	0.99305	0.998796	0.00546

Table S12: Summary of 98 drugs with different estimated IC50 value between two PANoptosis-related risk groups

Higher IC50 value	Drug
Low-risk group	Bleomycin, Cisplatin, Etoposide, Gemcitabine, Lapatinib, Methotrexate, Paclitaxel, Pazopanib, Rapamycin, Tipifarnib, A.443654, A.770041, AKT.inhibitor.VIII, AP.24534, AUY922, AZ628, AZD6482, AZD7762, Bexarotene, BI.2536, Bicalutamide, BMS.509744, BMS.536924, Bortezomib, Bryostatin.1, BX.795, Camptothecin, CCT018159, CEP.701, CGP.082996, CGP.60474, CHIR.99021, CMK, Cyclopamine, Cytarabine, Dasatinib, Docetaxel, Doxorubicin, Embelin, Erlotinib, FTI.277, GDC.0449, GDC0941, GSK269962A, GW843682X, JNK.Inhibitor.VIII, JW.7.52.1, KIN001.135, KU.55933, Metformin, MG.132, Midostaurin, Mitomycin.C, MK.2206, MS.275, NSC.87877, NU.7441, NVP.BEZ235, NVP.TAE684, Obatoclax.Mesylate, PAC.1, Parthenolide, PD.0325901, PF.02341066, PF.562271, PHA.665752, PLX4720, Pyrimethamine, RDEA119, RO.3306, Roscovitine, Salubrinal, SB.216763, Sorafenib, S.Trityl.L.cysteine, Thapsigargin, TW.37, Vinblastine, VX.680, WH.4.023, X17.AAG, XMD8.85, Z.LLNle.CHO, ZM.447439
High-risk group	ABT.263, BIRB.0796, BMS.509744, BMS.754807, CHIR.99021, GW.441756, IPA.3, Lenalidomide, LFM.A13, Methotrexate, Nutlin.3a, OSI.906, PD.0332991, SL.0101.1

Table S13: GO and KEGG enrichment analysis of DEGs between two PANoptosis-related risk groups in TCGA dataset

ONTOLOGY	Description	GeneRatio	p.adjust
BP	regulation of lymphocyte activation	124/2681	2.88E-09
BP	response to interferon-gamma	68/2681	1.55E-10
BP	response to tumor necrosis factor	67/2681	0.003715
BP	antigen processing and presentation	64/2681	1.28E-06
BP	JAK-STAT cascade	34/2681	0.039731
BP	macrophage activation	31/2681	9.52E-05
BP	leukocyte apoptotic process	27/2681	0.009674
BP	interleukin-10 production	25/2681	1.08E-06
BP	apoptotic cell clearance	15/2681	0.010092
CC	collagen-containing extracellular matrix	142/2834	7.65E-24
CC	endosome membrane	86/2834	0.056656
CC	cell projection membrane	81/2834	3.2E-05
CC	endocytic vesicle	71/2834	0.000113
CC	condensed chromosome	43/2834	0.084622
CC	MHC protein complex	18/2834	1.98E-09
CC	phagocytic vesicle membrane	18/2834	0.068436
CC	MHC class II protein complex	13/2834	5.36E-08
MF	channel activity	174/2667	7.06E-32
MF	receptor ligand activity	121/2667	1.34E-07
MF	cell adhesion molecule binding	107/2667	0.000891
MF	DNA-binding transcription activator activity	93/2667	0.003121
MF	cytokine activity	58/2667	0.000146
MF	immunoglobulin binding	10/2667	0.011871
MF	MHC protein complex binding	10/2667	0.016118
MF	peptide antigen binding	10/2667	0.066842
KEGG	ECM-receptor interaction	40/1284	3.02E-09
KEGG	Phagosome	54/1284	6.67E-08
KEGG	Cytokine-cytokine receptor interaction	80/1284	7E-06
KEGG	Complement and coagulation cascades	31/1284	3.22E-05
KEGG	Antigen processing and presentation	26/1284	0.000817
KEGG	Leukocyte transendothelial migration	32/1284	0.004253
KEGG	Proteoglycans in cancer	50/1284	0.006078
KEGG	PI3K-Akt signaling pathway	78/1284	0.007405
KEGG	Transcriptional misregulation in cancer	47/1284	0.007405
KEGG	MAPK signaling pathway	66/1284	0.009619
KEGG	Th1 and Th2 cell differentiation	26/1284	0.009649
KEGG	Th17 cell differentiation	28/1284	0.01919
KEGG	Primary immunodeficiency	12/1284	0.052009
KEGG	p53 signaling pathway	19/1284	0.071219

Table S14: GO and KEGG enrichment analysis of DEGs between two PANoptosis-related risk groups in CGGA dataset

ONTOLOGY	Description	GeneRatio	p.adjust
BP	antigen processing and presentation	125/3934	2.81E-27
BP	response to hypoxia	155/3934	2.15E-19
BP	regulation of apoptotic signaling pathway	164/3934	4.29E-17
BP	regulation of innate immune response	177/3934	7.36E-17
BP	response to interferon-gamma	95/3934	3.6E-15
BP	response to tumor necrosis factor	128/3934	5.25E-14
BP	regulation of Wnt signaling pathway	133/3934	2.01E-10
BP	response to radiation	148/3934	4.69E-08
BP	phagocytosis	111/3934	0.000243
BP	JAK-STAT cascade	54/3934	0.000541
BP	macrophage activation	32/3934	0.014053
BP	interleukin-10 production	20/3934	0.027263
CC	focal adhesion	250/4078	9.99E-71
CC	secretory granule lumen	173/4078	1.06E-37
CC	endoplasmic reticulum lumen	135/4078	1.54E-18
CC	lysosomal lumen	54/4078	2.5E-13
CC	phagocytic vesicle	62/4078	1.66E-10
CC	MHC class II protein complex	11/4078	0.000221
MF	cell adhesion molecule binding	226/3942	3.23E-28
MF	ubiquitin protein ligase binding	124/3942	8.35E-13
MF	NF-kappaB binding	17/3942	0.000696
MF	cytokine binding	47/3942	0.002659
MF	MHC protein complex binding	14/3942	0.00413
MF	damaged DNA binding	26/3942	0.011589
MF	peptide antigen binding	14/3942	0.032039
KEGG	Antigen processing and presentation	43/2122	8.43E-07
KEGG	Proteoglycans in cancer	84/2122	2.53E-05
KEGG	Apoptosis	60/2122	4.2E-05
KEGG	Mismatch repair	15/2122	0.000655
KEGG	p53 signaling pathway	34/2122	0.000869
KEGG	Th17 cell differentiation	45/2122	0.001459
KEGG	Ferroptosis	20/2122	0.007109
KEGG	Leukocyte transendothelial migration	44/2122	0.01054
KEGG	PI3K-Akt signaling pathway	116/2122	0.013046
KEGG	MAPK signaling pathway	96/2122	0.028287
KEGG	TNF signaling pathway	41/2122	0.03571
KEGG	Fc gamma R-mediated phagocytosis	42/2122	0.00112
KEGG	Transcriptional misregulation in cancer	67/2122	0.018002
